# Carotid Plaque‐Derived Small Extracellular Vesicles Mediate Atherosclerosis and Correlate With Plaque Vulnerability

**DOI:** 10.1002/mco2.70220

**Published:** 2025-05-19

**Authors:** Xin Xu, Taoyuan Lu, Yao Feng, Wenbo Cao, Dianwei Liu, Peng Gao, Yan Ma, Yabing Wang, Bin Yang, Yanfei Chen, Jian Chen, Ran Xu, Xinyu Wang, Lebin Chen, Yuanyuan Ji, Liqun Jiao

**Affiliations:** ^1^ Department of Neurosurgery Xuanwu Hospital, Capital Medical University Beijing China; ^2^ China International Neuroscience Institute (China‐INI) Beijing China; ^3^ Xuanwu Jinan Hospital Jinan China; ^4^ Department of Neurology Fuwai Hospital, National Center for Cardiovascular Diseases, Chinese Academy of Medical Sciences and Peking Union Medical College Beijing China; ^5^ Department of Stroke Center Central Hospital Affiliated to Shandong First Medical University Jinan China; ^6^ Hangzhou Dixiang Co. Ltd. Hangzhou China; ^7^ Department of Interventional Neuroradiology Xuanwu Hospital, Capital Medical University Beijing China

**Keywords:** asymptomatic carotid artery stenosis, atherosclerosis, biomarker, extracellular vesicle, microRNA, plaque vulnerability

## Abstract

Carotid plaque‐derived small extracellular vesicles (psEVs) offer insights into tissue‐ and disease‐specific pathobiology, but their roles in plaque vulnerability and their diagnostic potential remain unclear. Herein, we isolated psEVs from stable and vulnerable (intraplaque hemorrhage [IPH] or fibrous cap rupture [FCR]) plaques in patients with asymptomatic carotid artery stenosis (aCAS). Our findings demonstrated that psEVs alone were sufficient to induce inflammatory endothelial dysfunction in vitro and exacerbate atherogenesis in ApoE‐deficient mice. MicroRNA sequencing of psEVs (sequencing cohort, *n* = 18) identified 21 differentially expressed microRNAs (DEmiRNAs) distinguishing stable and vulnerable plaques, and 41 DEmiRNAs differentiating IPH from FCR subtypes. Subsequent validation using qRT‐PCR and the High‐throughput nano‐bio chip integrated system for liquid biopsy system revealed that plasma‐derived sEV miR‐497‐5p, miR‐152‐3p, and miR‐204‐5p effectively differentiated stable plaques from vulnerable plaques, while miR‐23a‐3p and miR‐143‐5p further distinguished IPH from FCR subtypes, in *both* the discovery cohort (n = 178) and an independent external cohort (n = 82). Mechanistic investigations identified miR‐497‐5p as a key mediator of vulnerable psEVs' proinflammatory and proatherogenic effects through directly targeting atheroprotective uncoupling protein 2 (UCP2). These findings highlight the roles of psEVs in atherogenesis and plaque vulnerability, providing valuable insights for risk stratification and therapeutic decision‐making in aCAS patients.

## Graphical abstract

We profiled the differences in miRNA expression of plaque‐derived small extracellular vesicles (psEVs) from stable and vulnerable plaques of patients with asymptomatic carotid artery stenosis (aCAS), and identified five plasma‐derived sEV miRNAs as potential biomarkers for diagnosing plaque vulnerability. miR‐497‐5p was found to mediate the proinflammatory and proatherogenic effects of vulnerable psEVs by directly targeting atheroprotective uncoupling protein 2 (UCP2).

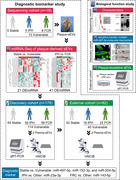

## 1 Introduction

Asymptomatic carotid artery stenosis (aCAS) is a significant risk factor for ischemic stroke [1, 2]. Current clinical guidelines lack consensus on the optimal management strategy for aCAS, with uncertainty surrounding the benefits of prophylactic revascularization compared with the best medical treatment alone [1, 2, 3, 4]. Conventional wisdom suggests that the stroke risk is linked to plaque size and the degree of luminal narrowing, but this assessment is not consistently reliable in aCAS cases [3, 4, 5]. Our recent study, which enrolled 2719 patients, showed that aCAS patients undergoing revascularization had a higher rate of 1‐month complications compared with those with symptomatic CAS [6]. Growing evidence suggests that carotid plaque composition or vulnerability, rather than its size, is more responsible for ischemic cerebrovascular events [7, 8]. The morphological characteristics of a vulnerable plaque typically include a large lipid‐rich necrotic core, a thin or ruptured fibrous cap, an intraplaque hemorrhage (IPH), or an intraluminal thrombus [7, 8, 9, 10]. Amongst, fibrous cap rupture (FCR) and IPH have been extensively demonstrated as predominant substrates for atherothrombosis, making them major contributors to ipsilateral ischemic stroke [7, 8, 9, 10]. Multiple clinical and imaging characteristics, along with molecular biomarkers, have been identified as potential indicators for the risk of vulnerable plaque or ischemic stroke in patients with CAS [10, 11, 12, 13]. However, the exact mechanisms underlying the formation and progression of vulnerable plaque remain incompletely understood [8]. Additionally, there is a scarcity of clinically reliable noninvasive biomarkers for identifying patients with vulnerable aCAS [12, 13].

Extracellular vesicles (EVs) are cellular vesicles enclosed by phospholipid bilayers that are typically classified into multivesicular body‐derived exosomes (30–200 nm), membrane‐derived microvesicles (or microparticles; 100–1000 nm), and apoptotic bodies (1–5 µm) [14, 15]. However, technical limitations in biogenesis‐specific EV isolation methods have prompted the suggestion of classifying EVs as small (sEV, <200 nm; including both exosomes and small microvesicles) and large (lEV, >200 nm) EVs to enhance the characterization of EV properties [14]. EVs play a crucial role in intercellular communications by delivering bioactive molecules (e.g., nucleic acids, proteins, and lipids) to adjacent and distant cells, thus regulating both physiological functions and pathophysiological processes [14, 15, 16]. Compelling evidence indicates that microRNAs (miRNAs) encapsulated within EVs are stable in biofluids and possess diagnostic and prognostic values for atherosclerosis [17, 18, 19, 20]. Moreover, EVs of different cellular origins, such as endothelial cells (ECs), smooth muscle cells (SMCs), and macrophages, have been mechanistically linked to atherogenic processes, rendering them potential therapeutic targets [19, 20, 21]. However, mechanistic studies typically utilize EVs obtained from cell culture supernatants, and diagnostic and prognostic studies rely on EVs extracted from body fluids, which may introduce confounding variables that are challenging to stratify and may lack specificity to particular tissue microenvironments and disease states [16, 22, 23]. Recent evidence demonstrates that proteins from atherosclerotic plaques exhibited little correlation with either circulating EVs or plasma fractions [24]. Shen et al. [23] have also highlighted significant differences in both contents and biological functions between sEVs derived from tumor tissues and those from cell culture supernatants.

EVs present in the interstitial spaces between tissue cells referred to as tissue‐derived (Ti)‐EVs, have recently been isolated and characterized primarily in solid tumor tissues, as well as in brain and heart tissues [22, 25–28]. Previous studies have investigated the roles of large microparticles (termed lEVs in a more recent study [29]), extracted from carotid endarterectomy (CEA) specimens, in atherosclerosis pathogenesis [29, 30, 31, 32]. However, the isolation of these Ti‐EVs was achieved through grinding or homogenization which may disrupt cells to release intracellular granules and artificial vesicles, potentially contaminating the Ti‐EV preparations. Until recently, Blaser et al. [33]. enriched Ti‐EVs from human atherosclerotic plaques and calcified aortic valves using methods similar to those previously employed in tumor Ti‐EV studies, and demonstrated their roles in cardiovascular calcification in a tissue‐specific manner. Peng et al. [34] isolated plaque‐derived lEVs (plEVs) and sEVs (psEVs) from rat atherosclerotic models, demonstrating that psEVs, rather than plEVs, were responsible for mediating atherogenesis at distant locations. However, the involvement of human psEVs in vulnerable plaque formation and their potential diagnostic significance remain largely unexplored.

To address this critical knowledge gap, we developed a method for isolating plEVs and psEVs from carotid plaques of aCAS patients with a particular focus on delineating the biological functions and mechanisms of psEVs in atherosclerotic pathogenesis. Furthermore, we profiled miRNAs in psEVs to delve into the potential of plasma‐derived sEV miRNAs as diagnostic biomarkers for plaque vulnerability through a multicohort clinical study design.

## 2 Results

### 2.1 Isolation and Characterization of plEVs and psEVs

**FIGURE 1:**
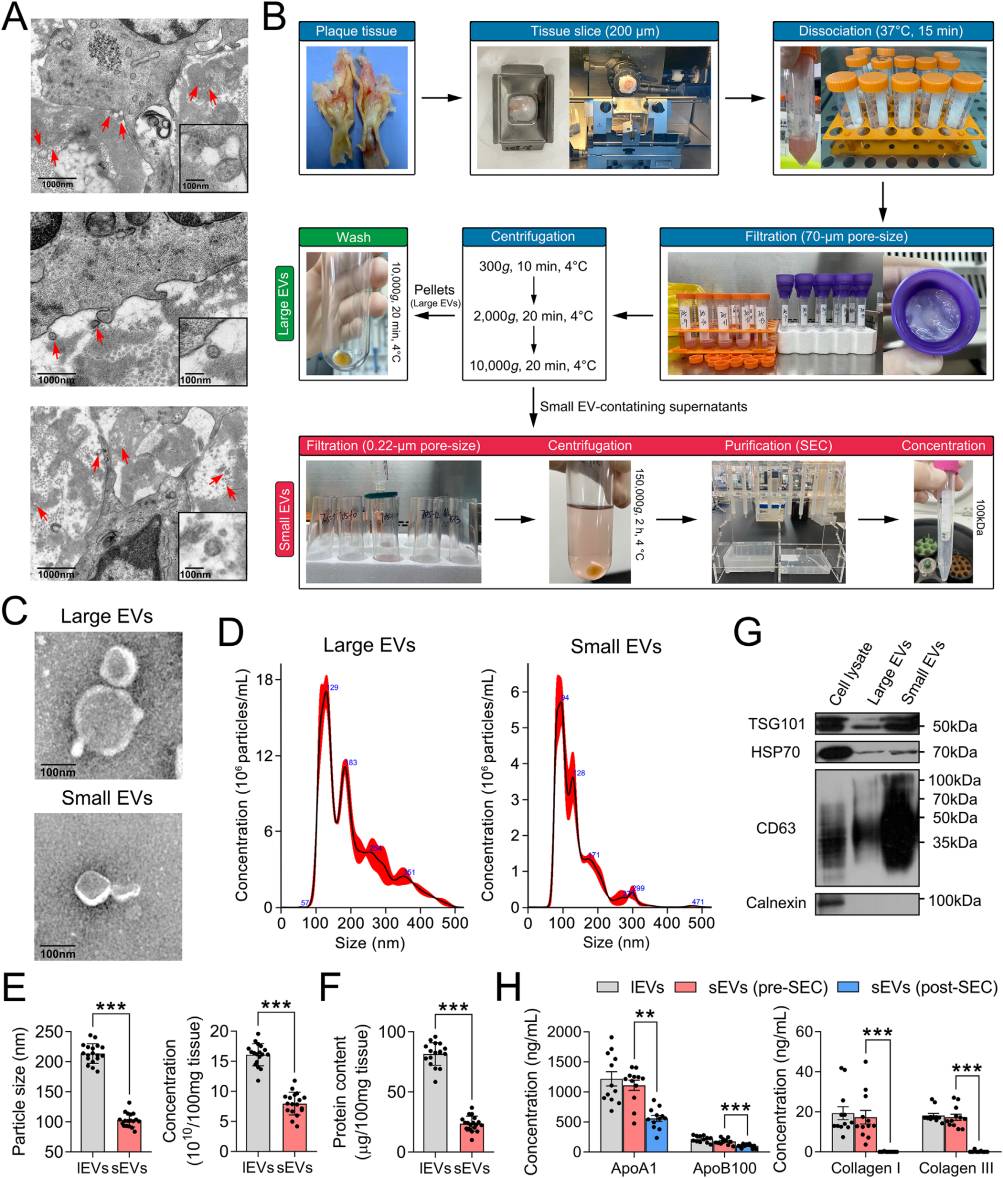
Isolation and characterization of plEVs and psEVs from aCAS patients in the sequencing cohort. (A) Representative TEM images of carotid plaque ultrathin sections showed the presence of Ti‐EVs within the extracellular spaces (red arrows). Scale bar = 100 or 1000 nm. (B) A schematic diagram illustrated the isolation of plEVs and psEVs. (C) Representative TEM images of the isolated plEVs and psEVs. Scale bar = 100 nm. (D–E) The size distribution of the isolated plEVs and psEVs was detected by NTA (D), and the particle size and concentration were obtained (E; *n* = 18/group). (F) The protein contents derived from the isolated plEVs and psEVs were quantified using a BCA kit (*n* = 18/group). G Representative western blotting bands of typical EV markers TSG101, HSP70, and CD63, and a negative marker calnexin (*n* = 5/group). (H) Non‐EV contaminants, including ApoA1, ApoB100, collagen I, and collagen III were measured using ELISA kits. Technical replicates = 3. Data were presented as the mean ± SD, and analyzed by *t*‐test or one‐way ANOVA followed by Bonferroni's multiple comparison test. **p < 0.01 and ***p < 0.001.

We first performed transmission electron microscopy (TEM) imaging of human carotid plaque ultrathin sections and observed the presence of Ti‐EVs within the extracellular spaces (Figure 1A). A multistep process involving liquid nitrogen snap‐freezing, 200 µm frozen sectioning, enzymatic digestion, ultracentrifugation, size‐exclusion chromatography (SEC) purification, and concentration was employed to enrich plEVs and psEVs (Figure 1B). Their typical characteristics were identified per the minimal information for studies of extracellular vesicles (MISEV2023) guidelines [14]. TEM imaging showed that both plEVs and psEVs exhibited morphologically similar cup‐shaped structures with completely or partially enclosed high‐density membranes (Figure 1C). Nanoparticle tracking analysis (NTA) displayed the vesicle size distribution, showing that plEVs were significantly larger (213.78 ± 16.41 vs. 103.04 ± 11.47 nm) and more concentrated (16.09 ± 1.84 × 10^10^ vs. 7.95 ± 1.9 × 10^10^ particles/100 mg tissue) than psEVs (Figure 1D,E). plEVs also exhibited a higher protein content (81.62 ± 9.4 vs. 23.65 ± 6.24 µg/100 mg tissue) as determined by bicinchoninic acid (BCA) assay. Western blotting analysis confirmed the presence of typical EV markers TSG101, HSP70, and CD63 in both plEVs and psEVs, while the negative marker calnexin remained undetectable (Figure 1G). Intriguingly, no significant differences in physical characteristics (size, concentration, and protein content) of both plEVs and psEVs were observed across different plaque subtypes (Table S1). Finally, we measured the levels of non‐EV contaminants, including apolipoprotein A1 (ApoA1; a contaminating marker of high‐density lipoprotein, HDL; accounts for ∼70% of total HDL protein content), ApoB100 (a contaminating marker of very low/low‐density lipoprotein, VLDL/LDL) [35], and extracellular matrix components collagen I and collagen III [36], using enzyme‐linked immunosorbent assay (ELISA). We found that, although the SEC method did not completely eliminate lipoproteins, the levels of ApoA1 (1.22 ± 0.23 %) and ApoB100 (0.32 ± 0.11 %) remained exceedingly low relative to the total protein contents of psEVs (Figure 1H). Furthermore, matrix proteins were properly removed, as evidenced by the minimal detection of collagen I and collagen III following SEC purification (Figure 1H), a result that was further confirmed by TEM (Figure S1). Taken together, these results suggest that Ti‐EVs are present in the extracellular spaces of human carotid plaques, and this multi‐step isolation process is effective in isolating both plEVs and psEVs.

### 2.2 psEVs Promoted Endothelial Inflammation In Vitro

**FIGURE 2:**
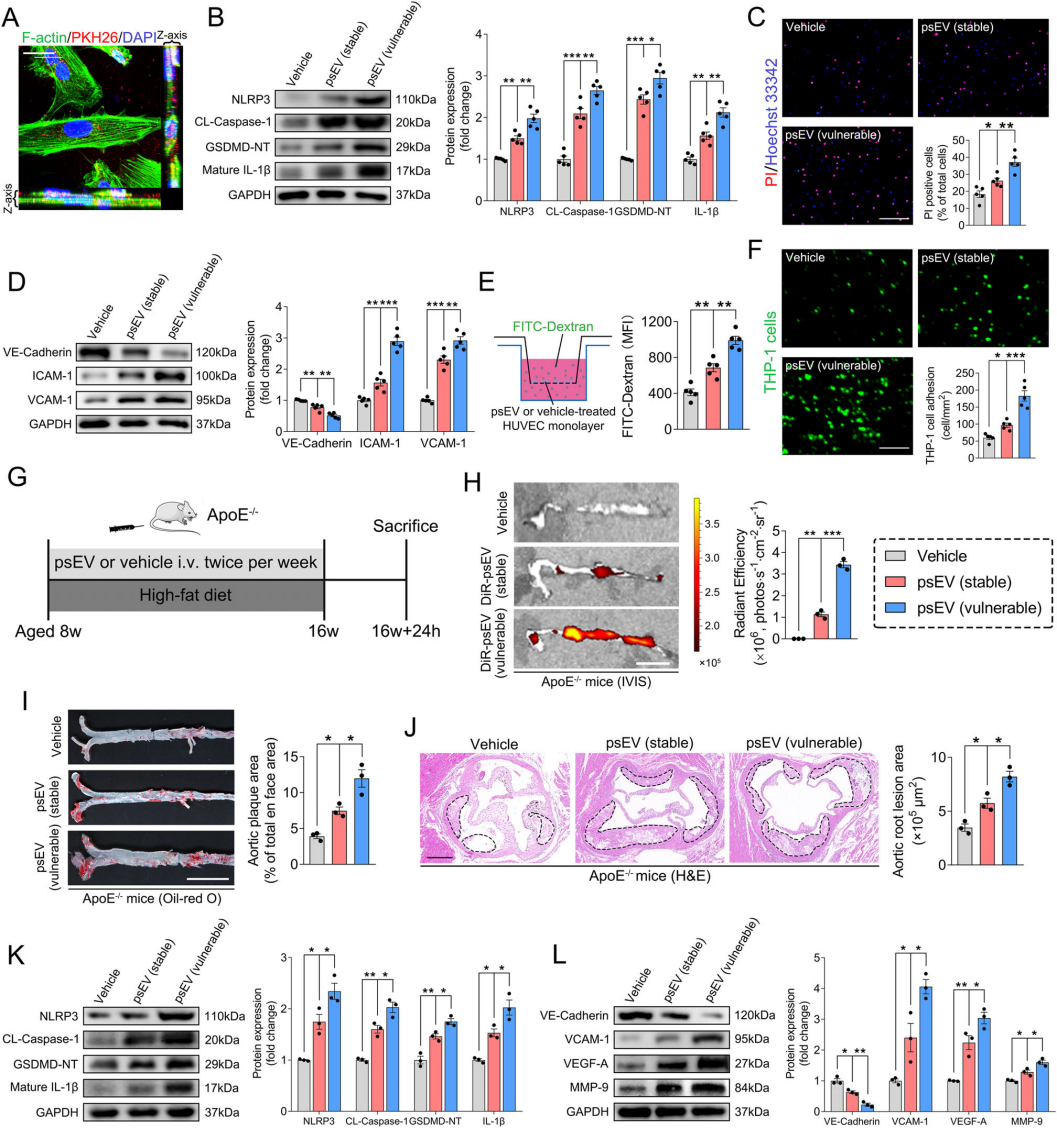
Effects of psEVs on endothelial inflammation in vitro and in vivo. (A) Representative 3D horizontal and vertical microphotograph showing PKH26‐labeled psEVs (red) taken up by HUVECs (F‐actin, green). Scale bar = 40 µm. (B) Representative western blotting bands and densitometric quantifications of NLRP3, CL‐caspase‐1, GSDMD‐NT, and mature IL‐1β in the treated HUVECs (*n* = 5/group). (C) Representative microphotographs of immunostaining and quantitative analysis of Hoechst 33342/PI‐positive cells (*n* = 5/group). Scale bar = 200 µm. (D) Representative western blotting bands and densitometric quantifications of VE‐cadherin, ICAM‐1, and ICAM‐1 in the treated HUVECs (*n* = 5/group). (E) Endothelial permeability was measured by FITC‐dextran (70 kDa) transendothelial leakage (*n* = 5/group). (F) Representative microphotographs and quantitative analysis of the adhesion of THP‐1 monocytes to treated HUVECs (*n* = 5/group). Scale bar = 200 µm. (G) Schematic of the experimental procedure to test the effect of psEVs on endothelial inflammation and atherogenesis using a high‐fat diet ApoE^−/−^ mouse model. i.v.: intravenous injection. (H) Representative ex vivo in vivo imaging system images of excised aorta and total radiant efficiency were calculated (*n* = 3/group). Scale bar = 1 cm. (I) Representative images of en face ORO staining of the entire aorta, and quantitative analysis of atherosclerotic plaque area (*n* = 3/group). Scale bar = 1 cm. (J) Representative microphotographs of H&E staining in cross‐sections on the aortic root, and quantitative analysis of atherosclerotic plaque area. Dashed lines delimited the plaque area. Scale bar = 400 µm. (K, L) Representative western blotting bands and densitometric quantifications of NLRP3, CL‐caspase‐1, GSDMD‐NT, mature IL‐1β, VE‐cadherin, VCAM‐1, VEGF‐A, and MMP‐9 in carotid artery tissues of ApoE^−/−^ mice (*n* = 3/group). Technical replicates = 3. Data were presented as the mean ± SD and were analyzed by one‐way ANOVA followed by Bonferroni's multiple comparison test. *p < 0.05, **p < 0.01, and ***p < 0.001.

Endothelial nucleotide‐binding oligomerization domain‐like receptor family pyrin domain containing 3 (NLRP3) inflammasome activation and subsequent canonical caspase‐1/gasdermin D (GSDMD)‐dependent pyroptosis contributes to vascular inflammation and atherogenesis [37, 38, 39, 40]. Our preliminary investigation focused on the biological functions of psEVs on endothelial inflammation in vitro. Filamentous (F)‐actin staining showed the internalization of PKH26‐labeled psEVs by human umbilical cord vein endothelial cells (HUVECs; Figure 2A). Western blotting analysis showed a significant increase in the levels of the NLRP3 inflammasome components NLRP3 and cleaved (CL)‐caspase‐1, the pyroptosis executor GSDMD‐N‐terminal (NT), and mature interleukin (IL)‐1β in HUVECs treated with either stable or vulnerable psEVs compared with those treated with vehicle controls (Figure 2B). Immunofluorescent staining showed that propidium iodide (PI)‐positive pyroptotic cells were remarkably elevated in HUVECs stimulated with psEVs from either stable or vulnerable plaques (Figure 2C). Additionally, western blotting analysis showed that both stable and vulnerable psEVs significantly downregulated vascular endothelial (VE)‐cadherin expression, while markedly upregulating the expressions of intercellular adhesion molecule 1 (ICAM‐1) and vascular cell adhesion molecule 1 (VCAM‐1) in HUVECs (Figure 2D), thus increasing endothelial permeability as measured by the transendothelial leakage of fluorescein isothiocyanate (FITC)‐dextran in a transwell system (Figure 2E) and enhancing the adhesion of THP‐1 monocytes to the treated HUVECs (Figure 2F). Notably, these changes were more pronounced in HUVECs stimulated with vulnerable psEVs (Figure 2B–F). EVs are membrane‐bound vesicles that are readily lysed by the detergent Triton X‐100, while non‐EV contaminants remain largely unaffected [41]. We subsequently pretreated vulnerable psEVs with Triton X‐100 and observed that vulnerable psEV‐induced endothelial inflammation, as measured by the aforementioned experiments (Figure 2B,D–F), was completely abrogated after psEV lysis (Figure S2), suggesting that psEVs, rather than non‐EV components, were responsible for mediating endothelial inflammation. Taken together, these results suggest that psEVs are sufficient to trigger endothelial inflammation, with vulnerable psEVs displaying stronger proinflammatory effects.

### 2.3 psEVs Promoted Endothelial Inflammation and Atherogenesis in Apolipoprotein E Knockout (ApoE^−/−^) Mice

We next fed ApoE^–/–^ mice with a high‐fat diet for 8 weeks to induce atherosclerosis [40]. TEM imaging of carotid plaque ultrathin sections showed the presence of Ti‐EVs within the extracellular spaces (Figure S3). Concurrent with the high‐fat diet, ApoE^−/−^ mice were intravenously injected with psEVs twice a week for 8 weeks to evaluate their impact on endothelial inflammation and atherogenesis on the atherosclerosis background (Figure 2G) [40]. Ex vivo imaging using the in vivo imaging system showed preferential accumulation of DiR‐labeled psEVs at aortic atherosclerotic lesions, with vulnerable psEVs exhibiting a higher targeting capacity (Figure 2H). En face, Oil Red O (ORO) staining of the entire aortas (Figure 2I) and hematoxylin–eosin (H&E) staining of aortic root cross‐sections (Figure 2J) showed significantly increased atherosclerotic lesion areas in mice treated with either stable or vulnerable psEVs compared with vehicle saline‐treated controls, with the most extensive plaques observed in mice treated with vulnerable psEVs. Subsequently, we detected the protein levels of endothelial inflammatory markers in carotid artery tissues and observed that the levels of NLRP3, CL‐caspase‐1, GSDMD‐NT, mature IL‐1β, and VCAM‐1 were significantly upregulated, while VE‐cadherin was significantly downregulated in both stable and vulnerable psEV‐treated ApoE^−/−^ mice, particularly prominent in those infused with vulnerable psEVs (Figure 2K,L). Vascular endothelial growth factor (VEGF; associated with intraplaque neovascularization and vascular permeability, etc.) and matrix metalloproteinase‐9 (MMP‐9; associated with inflammation and extracellular matrix degradation, etc.) are critical factors that contribute to plaque instability and rupture [42, 43]. We observed that the protein levels of VEGF‐A and MMP‐9 were significantly upregulated in carotid artery tissues of ApoE^−/−^ mice treated with either stable or vulnerable psEVs, with particularly pronounced increases noted in those treated with vulnerable psEVs (Figure 2L). Taken together, these findings suggest that psEVs play a role in aggravating endothelial inflammation and atherogenesis, with vulnerable psEVs exhibiting particularly pronounced effects, underscoring the involvement of psEVs in atherosclerosis progression and plaque instability.

### 2.4 Alterations of the miRNA Expression Profiles in psEVs

**FIGURE 3:**
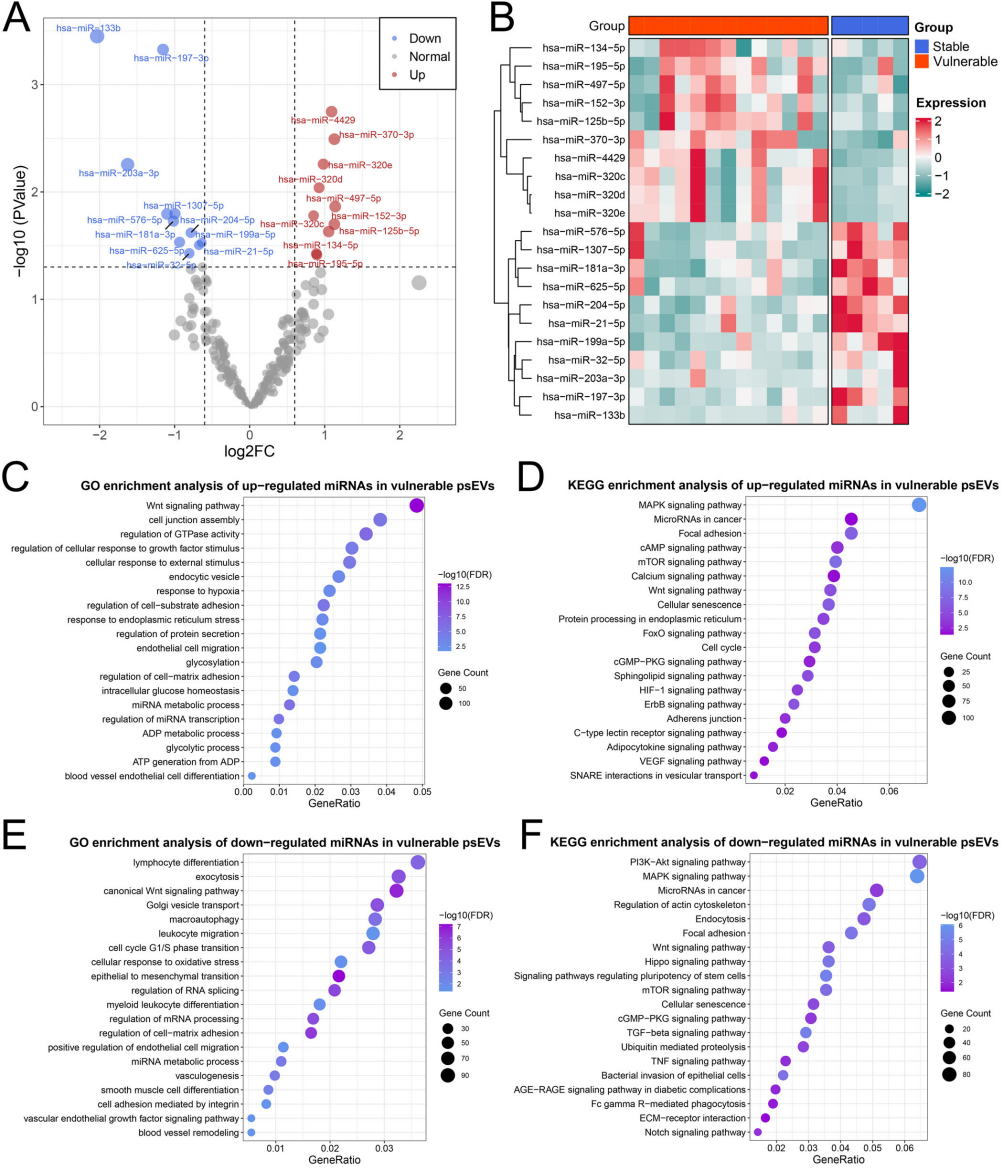
Bioinformatic analysis of miRNA sequencing of 13 vulnerable psEVs (5 IPH and 8 FCR) compared with five stable psEVs in the sequencing cohort. (A) Volcano plot of the DEmiRNAs between vulnerable and stable psEVs. Red and blue dots represent significantly upregulated and downregulated DEmiRNAs in vulnerable psEVs, respectively. (B) Heatmap of the normalized expression profiles of these DEmiRNAs. (B–F) GO and KEGG enrichment analyses for the target genes of the upregulated (C, D) and downregulated (E, F) DEmiRNAs.

**FIGURE 4:**
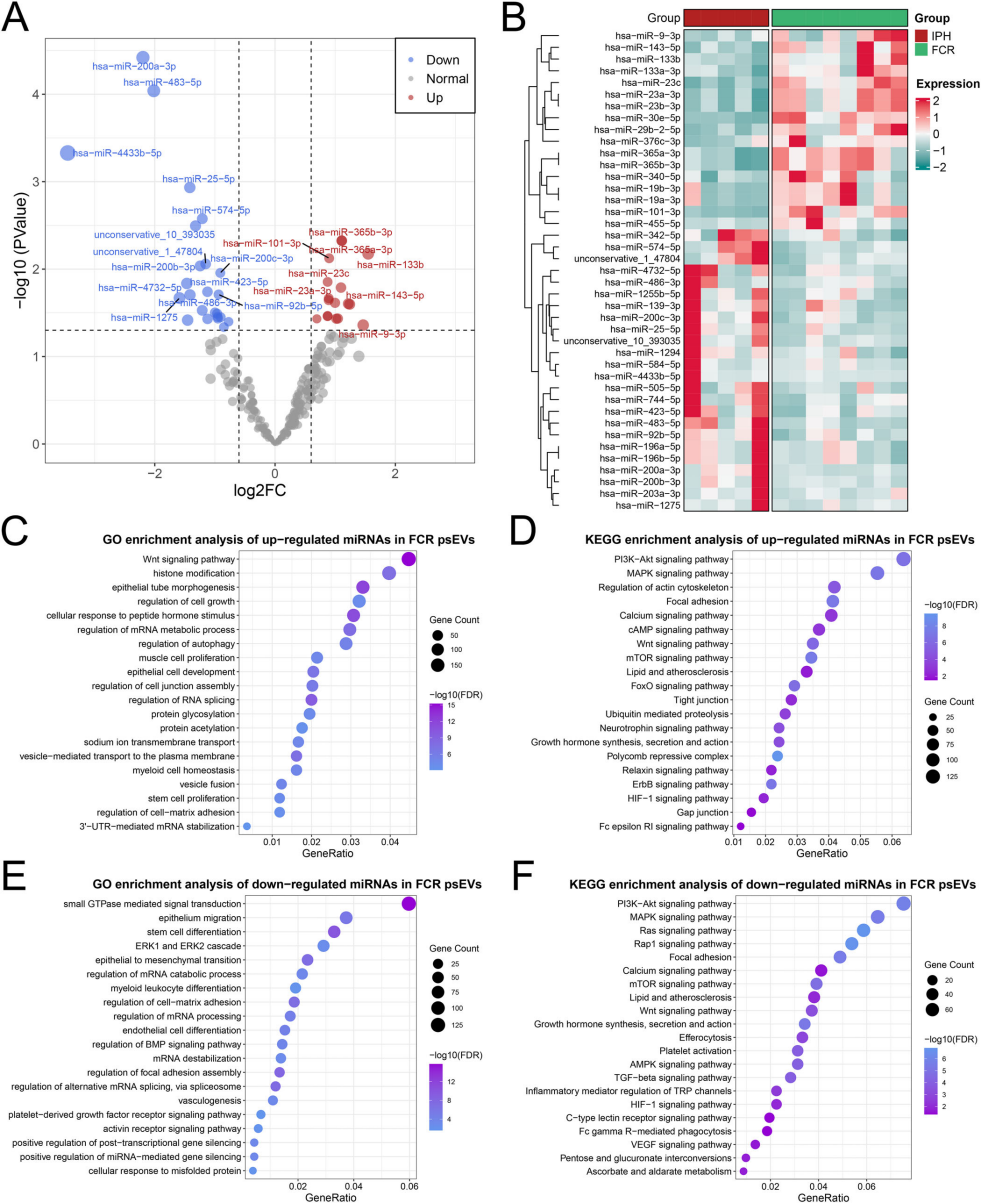
Bioinformatic analysis of miRNA sequencing of psEVs isolated from 8 FCR or 5 IPH plaques in the sequencing cohort. (A) Volcano plot of the DEmiRNAs between psEVs from IPH and FCR plaques. Red and blue dots represent significantly upregulated and downregulated DEmiRNAs in the FCR group, respectively. (B) Heatmap of the normalized expression profiles of these DEmiRNAs. (C–F) GO and KEGG enrichment analyses for the target genes of the upregulated (C, D) and downregulated (E, F) DEmiRNAs.

We further profiled miRNAs, a major class of mediators involved in the biological activities of sEVs, through microRNA sequencing (miRNA‐seq) on psEVs extracted from 18 aCAS patients (5 stable, 5 IPH, and 8 FCR) in the sequencing cohort (Table S2). Following quality control and miRNA annotation, a total of 2036 miRNAs (1,671 known and 365 novel) were identified. To ensure analytical robustness, 298 miRNAs with average transcripts per million (TPM) >10 were selected for subsequent analyses. Comparative analysis between 5 stable and 13 vulnerable psEVs identified 21 differentially expressed miRNAs (DEmiRNAs; 10 upregulated and 11 downregulated; Figure 3A,B; Table S3). Gene Ontology (GO) and Kyoto Encyclopedia of Genes and Genomes (KEGG) enrichment analyses suggested that the potential target genes of the 10 upregulated DEmiRNAs were significantly enriched in pathways related to cell signal transduction (e.g., Wnt signaling pathway and regulation of GTPase activity), RNA regulation (e.g., miRNA metabolic process, and regulation of miRNA transcription), stress response (e.g., response to endoplasmic reticulum stress, protein processing in endoplasmic reticulum, and response to hypoxia), energy metabolism (e.g., glycolytic process and ADP metabolic process), and cell junction (Figure 3C,D). The potential target genes of the 11 downregulated DEmiRNAs were mainly associated with immune activation (e.g., myeloid leukocyte differentiation and leukocyte migration), RNA processing (e.g., regulation of mRNA processing and regulation of RNA splicing), vasculogenesis, and cell cycle (Figure 3E,F). Further comparisons identified 17 upregulated miRNAs and 24 downregulated miRNAs in psEVs from FCR plaques relative to those from IPH plaques (Figure 4A,B; Table S3). GO and KEGG enrichment analyses suggested that the predicted target genes of the 17 upregulated DEmiRNAs were mainly enriched in pathways related to cell proliferation (e.g., regulation of cell growth, muscle cell proliferation, epithelial cell development, and regulation of actin cytoskeleton), RNA regulation (e.g., regulation of RNA splicing, regulation of mRNA metabolic process, and 3'UTR‐mediated mRNA stabilization), cell junction, and lipid and atherosclerosis (Figure 4C,D). While the potential target genes of the 24 downregulated DEmiRNAs were mainly linked to RNA regulation (e.g., regulation of alternative mRNA splicing via spliceosome and mRNA destabilization), coagulation (e.g., platelet activation and platelet‐derived growth factor receptor signaling pathway), focal adhesion, and angiogenesis (Figure 4E,F). The spreadsheets of miRNA‐seq analysis and bioinformatic analysis are listed in Table S4.

### 2.5 Plasma‐Derived sEV miRNAs Serve as Potential Diagnostics Biomarkers for Plaque Vulnerability

**FIGURE 5:**
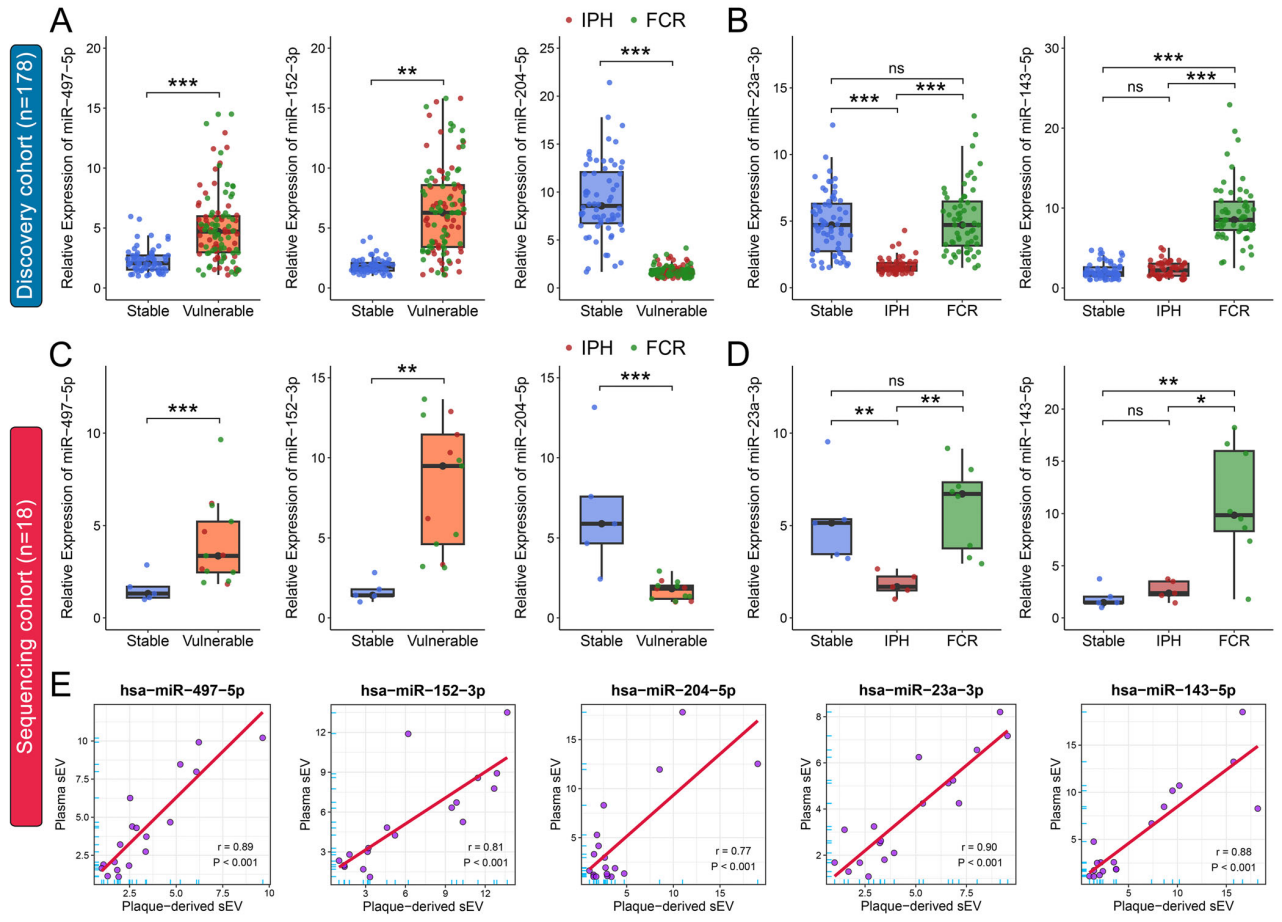
The expression levels of identified DEmiRNA candidates in plasma‐derived sEVs as detected by qRT‐PCR. (A–D) The relative expression of plasma‐derived sEV miR‐497‐5p, miR‐152‐3p, miR‐204‐5p, miR‐23a‐3p, and miR‐143‐5p in aCAS patients with stable (blue) or vulnerable (IPH: red; FCR: green) plaques in both the discovery cohort (*n* = 178, 64 stable, 55 IPH, 59 FCR; A, B) and the sequencing cohort (*n* = 18, 5 stable, 5 IPH, 8 FCR; C, D). (E) Pearson correlation coefficient analyses assessing correlations between DEmiRNAs from psEVs and those from plasma‐derived sEVs in the sequencing cohort (*n* = 18). Technical replicates = 3. Data were presented as the mean ± SD and were analyzed by *t*‐test or one‐way ANOVA followed by Bonferroni's multiple comparison test. **p < 0.05 and ***p < 0.001. ns: not significant.

Based on the candidate DEmiRNAs, we delved into the potential of plasma‐derived sEV miRNAs as indicators of plaque vulnerability. A discovery cohort of 178 aCAS patients (64 stable, 55 IPH, and 59 FCR; Table S5) was enrolled, their plasma‐derived sEVs were isolated, and the expressions of the candidate DEmiRNAs were analyzed using quantitative reverse transcription polymerase chain reaction (qRT‐PCR). We found that miR‐497‐5p and miR‐152‐3p were significantly upregulated, while miR‐204‐5p was significantly downregulated in plasma‐derived sEVs from aCAS patients with vulnerable (FCR or IPH) plaques relevant to those with stable plaques (Figure 5A). Furthermore, plasma‐derived sEV miR‐23a‐3p was significantly downregulated in patients with IPH plaques compared with those with stable or FCR plaques, while miR‐143‐5p was significantly upregulated in patients with FCR plaques compared with those with stable or IPH plaques (Figure 5B). No statistically significant differences of the other candidate DEmiRNAs in plasma‐derived sEVs among different plaque subtypes were observed, as detailed in Table S6.

Subsequently, we conducted a paired qRT‐PCR analysis on psEVs and plasma‐derived sEVs obtained from the same patient in the sequencing cohort (*n* = 18; Table S2). The diagnostic value of these five plasma‐derived sEV miRNAs in assessing plaque vulnerability was confirmed (Figure 5C,D), and we observed strong correlations between the five identified miRNAs from psEVs and plasma‐derived sEVs (Figure 5E). Finally, we isolated plaque‐exudative sEVs from a prospective external cohort of 82 aCAS patients (42 stable, 18 IPH, and 22 FCR; Table S7), using the explant culture method [44]. Human carotid plaques were sectioned into 200 µm slices and cultured in DMEM/F12 medium for 12 h, after which the released sEVs in the culture medium were enriched and characterized by TEM, NTA, and western blotting assays (Figure S4A–C). The mean size of plaque‐exudative sEVs was 115.17 ± 16.4 nm, with a concentration of 4.58 ± 1.88 × 10^7^ particles per 100 mg tissue. We then measured the miRNA expressions in plaque‐exudative sEVs by qRT‐PCR analysis and observed that the expression trends of the five identified miRNAs in sEVs released into the culture medium were consistent with those in psEVs (Figure S4D,E), suggesting the release of psEVs from plaques into the circulation. Taken together, we identify five plasma‐derived sEV miRNAs that may originate from plaques and can serve as potential biomarkers for plaque vulnerability.

### 2.6 Validation of the Diagnostics Values of Plasma‐Derived sEV miRNAs for Plaque Vulnerability by HNCIB System

**FIGURE 6:**
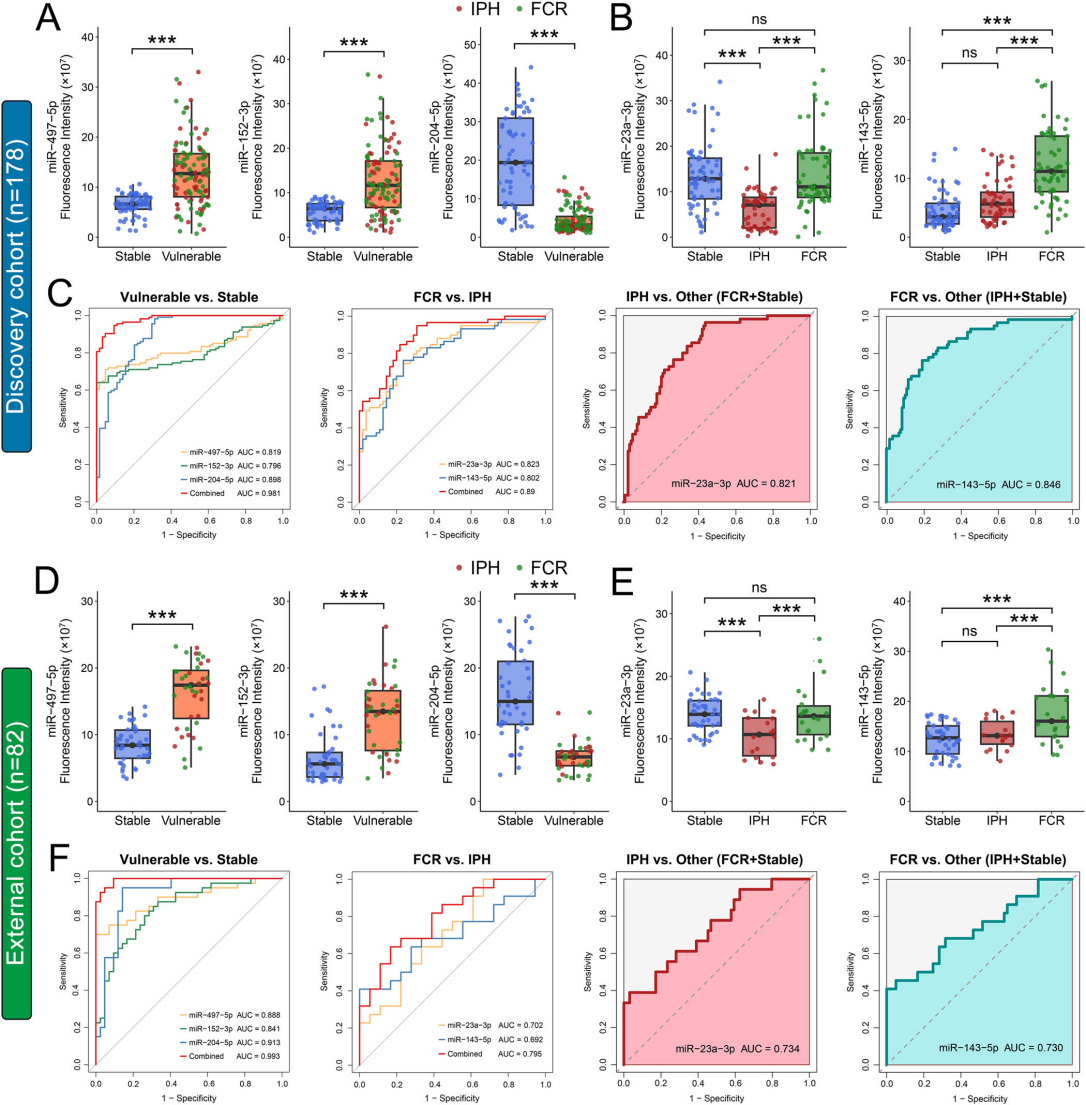
The expression levels of identified DEmiRNA candidates in plasma‐derived sEVs as detected by the HNCIB system. (A, B) The relative expression of plasma‐derived sEV miR‐497‐5p, miR‐152‐3p, miR‐204‐5p, miR‐23a‐3p, and miR‐143‐5p in aCAS patients with stable (blue, *n* = 64) or vulnerable (IPH: red, *n* = 55; FCR: green, *n* = 59) plaques in the discovery cohort. (C) ROC analyses for the plasma‐derived sEV DEmiRNAs to predict plaque vulnerability in the discovery cohort (*n* = 178). (D, E) The relative expression of plasma‐derived sEV miR‐497‐5p, miR‐152‐3p, miR‐204‐5p, miR‐23a‐3p, and miR‐143‐5p in aCAS patients with stable (blue, *n* = 42) or vulnerable (IPH: red, *n* = 18; FCR: green, *n* = 22) plaques in the external cohort. (F) ROC analyses for the plasma‐derived sEV DEmiRNAs to predict plaque vulnerability in the external cohort (*n* = 82). Technical replicates = 3. Data were presented as the mean ± SD and were analyzed by *t*‐test or one‐way ANOVA followed by Bonferroni's multiple comparison test. ***p < 0.001. ns: not significant.

High‐throughput nano‐bio chip integrated system for liquid biopsy (HNCIB) is a recently developed liquid biopsy system that allows for rapid and high‐throughput single‐EV analysis of both surface and cargo biomarkers (Figure S5A) [45]. This system exhibits high‐efficiency capture and enrichment, achieving an average particle density of ∼10^6^ particles/mm^2^ (Figure S5B). The mean fluorescent intensity (MFI) values detected by HNCIB validated significant differences in the expression levels of plasma‐derived sEV miR‐497‐5p, miR‐152‐3p, miR‐204‐5p, miR‐23a‐3p, and miR‐143‐5p among patients with different plaque subtypes, as observed in both sequencing cohort (*n* = 18; Figure S6A,B) and discovery cohort (*n* = 178; Figure 6A,B). In addition, the expression levels of these five miRNAs again exhibited a significant positive correlation between psEVs and plasma‐derived sEVs from the same patient in the sequencing cohort (Figure S6C). Further analysis using receiver operating characteristic (ROC) curves showed that the area under the curve (AUC) values for plasma‐derived sEV miR‐497‐5p, miR‐152‐3p, and miR‐204‐5p in identifying vulnerable plaques were 0.819 [95% confidence interval (CI), 0.757–0.881], 0.796 (95% CI, 0.731–0.86), and 0.898 (95% CI, 0.847–0.948), respectively (Figure 6C). When these three miRNAs were combined into a panel, the resulting AUC increased to 0.981 (95% CI, 0.968–0.995) (Figure 6C). Additionally, plasma‐derived sEV miR‐23a‐3p and miR‐143‐5p effectively differentiated IPH and FCR plaque subtypes with AUCs of 0.823 (95%CI, 0.746–0.899) and 0.802 (95%CI, 0.722–0.883), respectively (Figure 6C). The combination of these two miRNAs in a panel further improved the AUC to 0.89 (95% CI, 0.832–0.948). Furthermore, we evaluated the ability of miR‐23a‐3p and miR‐143‐5p to specifically detect vulnerable plaque subtypes. The AUC for plasma‐derived sEV miR‐23a‐3p in distinguishing IPH from other plaques was 0.821 (95%CI, 0.759–0.883), while the AUC for plasma‐derived sEV miR‐143‐5p in distinguishing FCR from other plaques was 0.846 (95%CI, 0.785–0.907) (Figure 6C). To further validate the diagnostic accuracy of the five plasma‐derived sEV miRNAs, an independent external cohort of 82 aCAS patients (42 stable, 18 IPH, and 22 FCR; Table S7) was prospectively recruited. The HNCIB analysis confirmed significant differences in the expressions of plasma‐derived sEV miR‐497‐5p, miR‐152‐3p, miR‐204‐5p, miR‐23a‐3p, and miR‐143‐5p among patients with different plaque subtypes (Figure 6D,E). These differences were further corroborated by ROC analyses (Figure 6F), with all AUC values from the external cohort being comparable to those observed in the discovery cohort (Table S8). Taken together, these results suggest that plasma‐derived sEV miR‐497‐5p, miR‐152‐3p, and miR‐204‐5p have the potential as diagnostic biomarkers to distinguish between stable and vulnerable plaques, while plasma‐derived sEV miR‐23a‐3p and miR‐143‐5p can further distinguish IPH from FCR.

### 2.7 Vulnerable psEV‐Encapsulated miR‐497‐5p Promoted Endothelial Inflammation by Directly Targeting Uncoupling Protein 2 (UCP2) and Activating Reactive Oxygen Species (ROS)/Thioredoxin‐Interacting Protein (TXNIP)/NLRP3 Pathway

**FIGURE 7:**
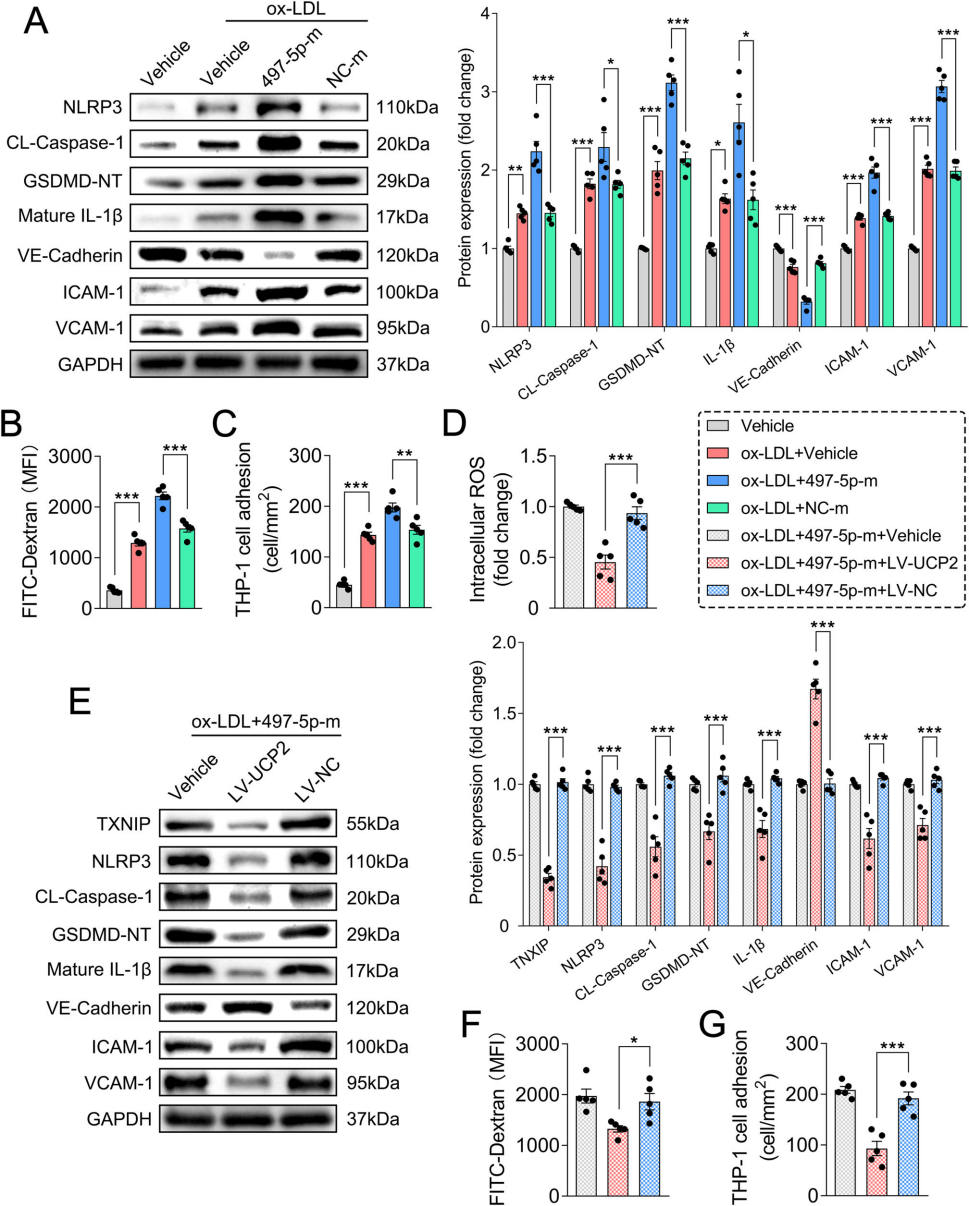
Effects of miR‐497‐5p on endothelial inflammation in ox‐LDL‐treated HUVECs. (A) Representative western blotting bands and densitometric quantifications of NLRP3, CL‐caspase‐1, GSDMD‐NT, mature IL‐1β, VE‐cadherin, ICAM‐1, and VCAM‐1 in the treated HUVECs (*n* = 5/group). (B) Endothelial permeability was measured by FITC‐dextran (70 kDa) transendothelial leakage (*n* = 5/group). (C) Quantitative analysis of the adhesion of THP‐1 monocytes to treated HUVECs (*n* = 5/group). (D) The treated HUVECs were collected and subjected to intracellular reactive ROS measurement (*n* = 5/group). (E) Representative western blotting bands and densitometric quantifications of NLRP3, CL‐caspase‐1, GSDMD‐NT, mature IL‐1β, VE‐cadherin, ICAM‐1, and VCAM‐1 in treated HUVECs (*n* = 5/group). Technical replicates = 3. Data were presented as the mean ± SD and were analyzed by one‐way ANOVA followed by Bonferroni's multiple comparison test. *p < 0.05, **p < 0.01, and ***p < 0.001. ns: not significant.

As sEVs exert biological functions by delivering cargo miRNAs into recipient cells [15], we selected miR‐497‐5p, one of the upregulated DEmiRNAs in vulnerable psEVs, to primarily investigate the functional molecules responsible for vulnerable psEV‐mediated endothelial inflammation. qRT‐PCR analysis confirmed that the expression levels of miR‐497‐5p in HUVECs treated with vulnerable psEVs were significantly increased compared with those treated with stable psEVs and vehicle controls (Figure S7A). Furthermore, the expression levels of miR‐497‐5p were increased in HUVECs treated with oxidized (ox)‐LDL, a well‐known atherogenic factor, in a dose‐ (25, 50, 100, and 200 µg/mL) and time‐ (12, 24, and 48 h) dependent manner (Figure S7B). Subsequently, by utilizing miR‐497‐5p mimics and inhibitors (Figure S7C), we evaluated the effects of miR‐497‐5p on endothelial inflammation in ox‐LDL‐stimulated HUVECs, which served as an in vitro atherosclerotic model. Compared with transfection with the negative control (NC) mimics, ox‐LDL‐stimulated HUVECs transfected with miR‐497‐5p mimics exhibited a significant increase in the protein levels of NLRP3, CL‐caspase‐1, GSDMD‐NT, IL‐1β, ICAM‐1, and VCAM‐1, a notable decrease in VE‐cadherin expression, along with increased endothelial permeability and THP‐1 cell adhesion (Figures 7A–C; Figure S7D).

**FIGURE 8:**
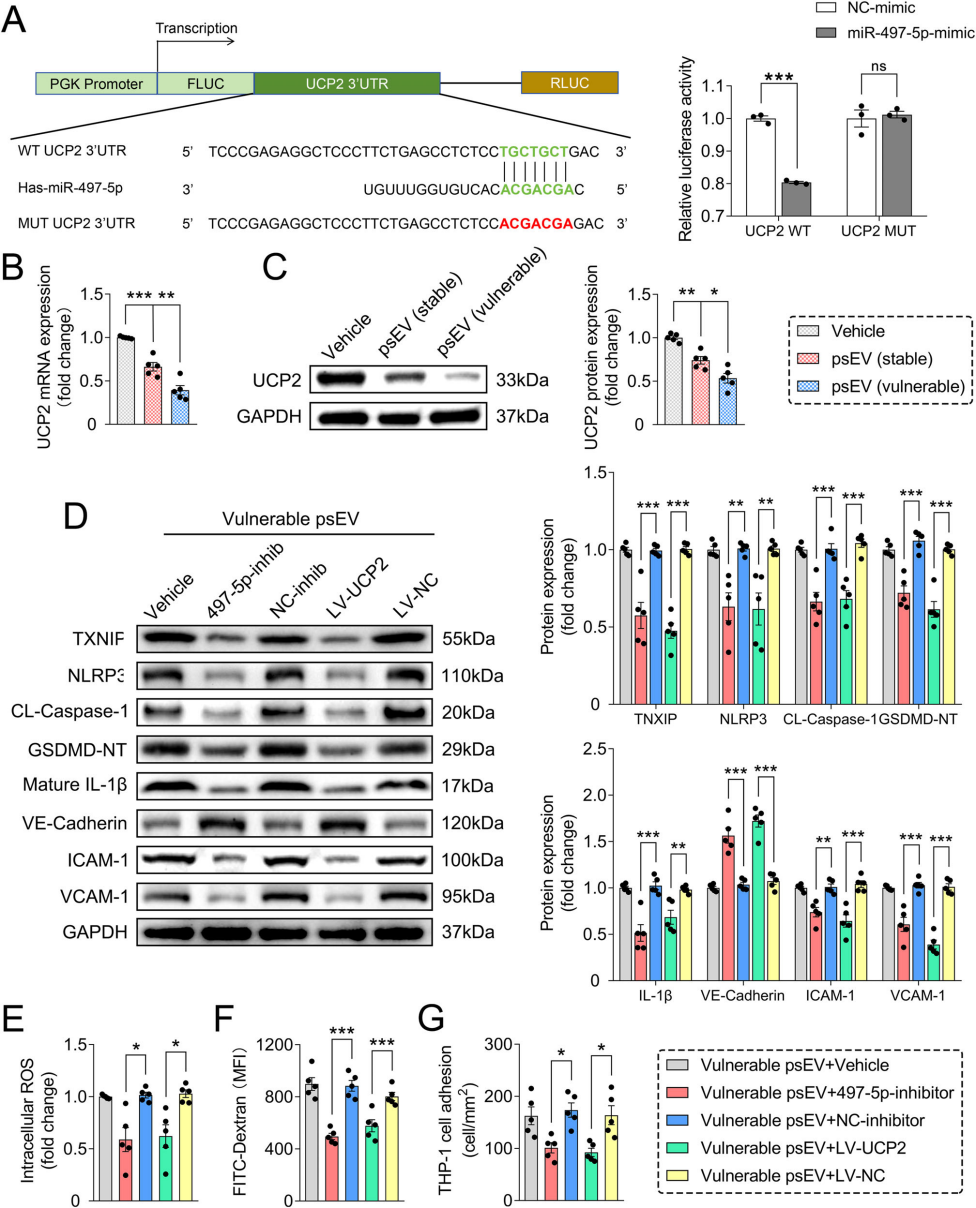
Vulnerable psEVs promoted endothelial inflammation partially through miR‐497‐5p delivery and UCP2 suppression. (A) The binding sites between miR‐497‐5p and the 3′UTR of UCP2 were predicted by an online tool TargetScan, and their binding relationships were verified using a dual‐luciferase reporter assay. (E) The mRNA and protein expression levels of UCP2 in vehicle or psEV‐treated HUVECs (n = 5/group). (D) Representative western blotting bands and densitometric quantifications of NLRP3, CL‐caspase‐1, GSDMD‐NT, mature IL‐1β, VE‐cadherin, ICAM‐1, and VCAM‐1 in the treated HUVECs (n = 5/group). (E) Endothelial permeability measured by FITC‐dextran (70 kDa) transendothelial leakage (*n* = 5/group). (F) Quantitative analysis of the adhesion of THP‐1 monocytes to treated HUVECs (*n* = 5/group). (G) The treated HUVECs were collected and subjected to intracellular reactive ROS measurement. Technical replicates = 3. Data were presented as the mean ± SD and were analyzed by one‐way ANOVA followed by Bonferroni's multiple comparison test. *p < 0.05, **p < 0.01, and ***p < 0.001. ns: not significant.

We further elucidate the molecular mechanism by which miR‐497‐5p participated in endothelial inflammation. Target genes of miR‐497‐5p were predicted using the online tool TargetScan (https://www.targetscan.org/), and a total of 1141 putative targets were predicted (Table S9). Given that miRNAs regulate gene expression through translational repression and/or mRNA deadenylation and decay [17]. We speculated that miR‐497‐5p mediated endothelial inflammation and atherosclerosis by inhibiting target genes associated with anti‐inflammatory and atheroprotective processes. Amongst, we focused on UCP2, a key mitochondrial antioxidant protein that has been reported to be protective against endothelial oxidative stress, inflammation, and atherosclerosis [46, 47, 48]. Previous evidence has also shown that UCP2 deficiency exacerbates ROS production to activate the NLRP3 inflammasome by facilitating the interaction between TXNIP and NLRP3 [49]. We found that both mRNA and protein levels of UCP2 were decreased in miR‐497‐5p mimic‐transfected HUVECs, while increased in miR‐497‐5p inhibitor‐transfected HUVECs (Figure S8A,B). The miR‐497‐5p mimic‐treated and ox‐LDL‐stimulated HUVECs exhibited increased ROS production and TXNIP expression, these effects were significantly attenuated after UCP2 overexpression using a lentivirus overexpression vector (Figures 7D,E; Figure S8C). Furthermore, the miR‐497‐5p‐mediated NLRP3/caspase‐1/GSDMD pathway and the resulting inflammatory endothelial dysfunction in ox‐LDL‐stimulated HUVECs was also significantly attenuated after UCP2 overexpression (Figures 7E–G; Figure S8D). To validate whether UCP2 is a target gene of miR‐497‐5p, we performed a dual‐luciferase reporter assay and identified the putative miR‐497‐5p binding site located at sites 9–15 of the UCP2 3′untranslated region (3′UTR), as predicted by TargetScan (Figure 8A). Luciferase vectors containing either wild‐type (WT) or mutated (MUT) UCP2 3’UTR were transfected into HEK293T cells, alongside either miR‐497‐5p mimics or NC mimics. The results showed that co‐transfection of miR‐497‐5p mimics with the WT UCP2 3’UTR vectors led to a decrease in luciferase activity, while no effect was observed when the miR‐497‐5p binding sites were mutated (Figure 8A), indicating that UCP2 was a direct target of miR‐497‐5p.

Finally, we observed that vulnerable psEV‐stimulated HUVECs exhibited a significant reduction in UCP2 expression at both mRNA and protein levels when compared with those treated with stable psEVs or vehicle controls (Figure 8B,C). Moreover, the activation of the ROS/TXNIP/NLRP3 pathway and the subsequent endothelial inflammation induced by vulnerable psEVs were notably mitigated upon co‐transfection with miR‐497‐5p inhibitors or UCP2 overexpression lentiviruses (Figures 8D–G; Figure S8E). Taken together, these results suggest that vulnerable psEVs promote endothelial inflammation and atherosclerosis partially through miR‐497‐5p delivery and UCP2 suppression to modulate the ROS/TXNIP/NLRP3 pathway.

## 3 Discussion

In this study, we successfully isolated plEVs and psEVs from carotid plaques of aCAS patients. Functional characterization revealed that psEVs were sufficient to induce inflammatory endothelial dysfunction in vitro and exaggerate endothelial inflammation and atherogenesis in ApoE^−/−^ mice, with vulnerable psEVs displaying stronger proinflammatory and proatherogenic effects. Subsequently, we reported differences in miRNA profiles of psEVs from different plaque subtypes. Based on the identified DEmiRNAs, we further analyzed sEVs from patients’ plasma samples and identified five plasma‐derived sEV miRNAs (miR‐497‐5p, miR‐152‐3p, miR‐204‐5p, miR‐23a‐3p, and miR‐143‐5p) as potential diagnostic biomarkers for plaque vulnerability. Finally, mechanistic investigations revealed that vulnerable psEVs promoted endothelial inflammation and atherosclerosis partially through miR‐497‐5p delivery and UCP2 suppression to activate the ROS/TXNIP/NLRP3 pathway.

Ti‐EVs offer advantages over biofluid‐ or culture media‐derived EVs by accurately reflecting tissue‐ and disease‐specific biological activities. They can originate from various cell types within tissues, move within the intercellular matrix, and function locally or enter the circulation to affect remote organs. Consequently, Ti‐EVs represent promising candidates for diagnosis, prognosis, and therapeutic interventions [22, 25, 27]. However, current Ti‐EV isolation methodologies face critical challenges, with the most challenging step being assurance that the isolated EVs originate from the extracellular space rather than result from tissue homogenization [14, 16, 22, 27]. Several studies have enriched large microparticles (referred to as lEVs in a recent study [29]) and demonstrated their proatherogenic effects. These vesicles are extracted by mincing tissues, which may introduce contaminations and mechanic injuries to tissue cells [29, 30, 31, 32]. Recently, Blaser et al. [33]. reported the enrichment of Ti‐EVs from human‐diseased cardiovascular tissues by roughly cubing, collagenase digestion, and density gradient ultracentrifugation, similar to previous methods in solid tumor studies. In the present study, we successfully isolated plEVs and psEVs and confirmed that the characteristics of these Ti‐EVs met the MISEV2023 criteria [14]. We employed liquid nitrogen snap‐freezing and 200‐µm frozen sectioning, which offers several advantages including specimen preservation, repeatability, standardization, time efficiency, better control over the degree of enzymatic digestion, and increased EV yields [50]. Furthermore, the combination of ultracentrifugation and SEC has been demonstrated to enhance both the purity and yield of the isolated EVs and to decrease protein contamination (e.g., extracellular matrix protein) [51]. It is important to note that, although minimal lipoproteins were co‐isolated (Figure 1H), the current isolation method cannot completely eliminate them, a finding that is consistent with previous reports [52]. However, through EV lysis using Triton‐X 100, we have demonstrated that the psEVs, rather than non‐EV components, exerted biological functions (Figure S2), which underscores the efficacy of the current EV isolation method. In addition, this method cannot distinguish between different psEV subpopulations, such as exosomes and small microvesicles. To address these limitations, combining SEC with an iodixanol‐based density gradient centrifugation method may provide a potential solution worthy of further investigation [52, 53].

Endothelial dysfunction plays an essential role in the initiation and progression of atherosclerosis [38]. Our preliminary investigation focused on the biological functions of psEVs on vascular ECs. We demonstrated that psEVs had the ability to activate the endothelial NLRP3 inflammasome and trigger the subsequent pyroptosis, leading to endothelial dysfunction that is characterized by the disruption of adherens junctions, loss of vascular integrity, increase in vascular permeability, upregulation of adhesion molecules, and accumulation of proinflammatory leukocytes within the growing atherosclerotic plaque [37, 38]. Moreover, we found that psEVs aggravated in vivo endothelial inflammation and atherogenesis in a high‐fat diet ApoE^−/−^ mouse model of atherosclerosis. Notably, psEVs from vulnerable plaques exhibited a higher targeting capacity toward atherosclerotic plaques and facilitated more severe atherosclerosis pathogenesis, thus highlighting psEVs in atherogenesis and plaque instability. In support of our observation, Peng et al. [34] found that psEVs from rat atherosclerotic plaques, rather than plEVs, transferred miR‐23a‐3p to promote endothelial inflammation and monocyte recruitment, ultimately inducing atherogenesis. Additionally, large microparticles obtained from human atherosclerotic lesions extracted through mincing and centrifugation have been implicated in multiple proatherogenic processes, including endothelial dysfunction, inflammation, angiogenesis, and SMC migration [29, 30, 31, 32]. Our further miRNA‐seq analysis identified 21 DEmiRNAs between stable and vulnerable psEVs, and subsequent analysis of psEVs from IPH and FCR plaques revealed 41 DEmiRNAs. Related bioinformatic analysis provides insights into the potential mechanisms underlying the dynamic evolution of plaques toward various pathological phenotypes, warranting further investigation.

miRNAs are major bioactive components within sEVs that accurately represent disease states [14, 16]. They are well protected from degradation by the lipid bilayer membrane, stable in biofluids, and detectable through high‐throughput techniques [14, 16, 17]. A study by Jansen et al. [54] demonstrated that miRNAs encapsulated in EVs, but not free miRNA, predicted the occurrence of cardiovascular events in patients with stable coronary artery disease. In the present study, we delved deeper into the expression of DEmiRNAs in plasma‐derived sEVs to identify clinically valuable biomarkers. Ultimately, plasma‐derived sEV miR‐497‐5p, miR‐152‐3p, miR‐204‐5p, miR‐23a‐3p, and miR‐143‐5p were identified as potential diagnostic biomarkers for plaque vulnerability. Previous studies have also suggested several plasma sEV‐derived miRNAs as markers for the diagnosis or risk stratification of atherosclerosis but did not definitively establish whether their origins are from plaque derivatives [17, 18]. In this study, we established strong correlations between the five identified miRNAs from psEVs and plasma‐derived sEVs obtained from the same patient (Figures 5E; Figure S6C). Moreover, we isolated plaque‐exudative sEVs using the explant culture method and confirmed that the expression patterns of the five miRNAs in sEVs released into the culture medium were consistent with those in psEVs (Figure S4D,E). These findings may lend support to the hypothesis that the isolated psEVs originate from plaques and subsequently enter the circulation. However, theoretically, it is essential to consider another possibility: circulating sEVs may become trapped within plaques, thereby reflecting the cargo of sEVs present in circulation. Additionally, psEVs may originate from other diseased organs. For example, it has been reported that steatotic hepatocytes release sEVs that promote endothelial inflammation and atherogenesis [55, 56]. Hence, further investigation into the spatiotemporal distribution of psEVs is warranted.

Since sEVs exert biological functions by delivering cargo miRNAs into recipient cells [15]. In the present study, we specifically selected the upregulated miR‐497‐5p to investigate the functional molecules responsible for vulnerable psEV‐mediated endothelial inflammation and atherosclerosis. We confirmed that vulnerable psEVs could deliver miR‐497‐5p into treated HUVECs and that miR‐497‐5p overexpression aggravated endothelial inflammation in vitro. We then identified UCP2, an anion transporter protein located in the inner mitochondrial membrane, which serves as a key mitochondrial antioxidant protein. UCP2 expression is reduced in atherosclerotic plaques, and its atheroprotective function is likely mediated by decreasing mitochondrial potential to restrict ROS production, as well as by inhibiting inflammation and cell death [46, 47, 48]. Luo et al. [46]. reported that UCP2 acts as a mechanosensitive suppressor, with its expression being critical for the endothelial proinflammatory response and atherogenesis. In this study, we observed that miR‐497‐5p‐mediated inflammatory endothelial dysfunction in ox‐LDL‐stimulated HUVECs was attenuated following UCP2 overexpression, and confirmed that UCP2 was a direct target gene of miR‐497‐5p. Furthermore, we demonstrated that vulnerable psEVs promoted endothelial inflammation and atherosclerosis, partially through the delivery of miR‐497‐5p and subsequent suppression of UCP2, thereby activating the ROS/TXNIP/NLRP3 pathway, a well‐established mechanism of NLRP3 activation [57]. The involvement of these five miRNAs in atherosclerosis is also supported by previous reports. Shan et al. [58] demonstrated that miR‐497‐5p was increasingly upregulated as atherosclerosis progressed in ApoE^−/−^ mice. Lu et al. [59] observed increased miR‐497‐5p levels in ox‐LDL‐stimulated HUVECs, and found that miR‐497‐5p exacerbated ox‐LDL‐induced endothelial dysfunction via targeting *VEGFA* to activate the p38/MAPK pathway. miR‐152‐3p has been shown to aggravate vascular endothelial dysfunction under hypoxia [60], and bioinformatics analysis suggests it regulates *KRAS* to contribute to atherosclerosis pathogenesis [61]. The expression levels of miR‐204‐5p are significantly lower in human atherosclerotic plaque tissues and blood samples compared with healthy controls [62], as well as in a rabbit model of carotid atherosclerosis and in vitro ox‐LDL‐stimulated HUVECs [63, 64]. Further mechanistic investigations revealed that miR‐204‐5p played a crucial role in the growth and migration of human SMCs by targeting MMP‐9 [62]. Guo et al. [65] demonstrated that the levels of miR‐23a‐3p were reduced in both in vitro and in vivo atherosclerotic models and that miR‐23a‐3p regulated proinflammatory and apoptotic pathways in atherogenesis by targeting proinflammatory *TNFAIP3*. miR‐143‐5p and its co‐transcribed cluster member miR‐145 are pivotal for vascular SMC differentiation and disease, but the specific role of miR‐143‐5p in atherosclerosis development remains to be addressed [66]. These findings enhance the understanding of the five plasma‐derived sEV miRNAs as potential biomarkers for plaque vulnerability.

This study also benefits from the integrated HNCIB system, which enables single‐EV capture and visualization with concurrent detection of both surface markers and intravesicular cargo components [45]. In the present study, the HNCIB analysis confirmed the effectiveness of the five miRNAs in diagnosing plaque vulnerability across sequencing and discovery cohorts, with further validation in a prospective external cohort. Notable characteristics of HNCIB technology include minimal sample requirement (∼90 µL plasma samples), high speed (∼6 h from sample acquisition to results), and high throughput capacity (up to 384 samples). Furthermore, the biochip module is designed with a clinically compatible form factor that enhances usability. This system facilitates rapid analysis and accurate interpretation of results through its fully automated data processing module, while the integration of deep learning algorithms enhances the precision and reliability of result interpretations [45]. Should future studies with larger sample sizes establish reference values for diagnosing plaque vulnerability, the HNCIB system is expected to make significant contributions to community health examination screenings for the early risk stratification of aCAS.

This study has several limitations that should be addressed in future investigations. First, the definition of vulnerable plaques focused exclusively on IPH and FCR, other pathological characteristics or a combination of various characteristics of vulnerable plaques require additional investigation. Second, in sequencing cohort, patients with vulnerable plaques exhibited a numerically higher frequency of diabetes and dyslipidemia (Table S2), both of which are common risk factors for plaque vulnerability [67], this may introduce bias in miRNA‐seq analysis and biological functional interpretation. Third, the sample size of the sequencing cohort was limited, which may reduce the statistical power to detect subtle differences. Additionally, although we recruited an external validation cohort, its sample size was also relatively small. Our study serves as a pilot and exploratory investigation that lays the foundation for larger, adequately powered, and prospective multicenter trials. Fourth, we specifically selected miR‐497‐5p to primarily investigate the molecular mechanisms underlying vulnerable psEV‐mediated endothelial inflammation, the pleiotropic effects of psEVs on various cell types within the plaque and their intercellular communication require further investigation. An integrative multi‐omics investigation of the composition and function of psEVs may provide valuable resources for comprehensive mechanistic studies. Fifth, the co‐isolation of non‐EV soluble proteins and lipoproteins may influence the detection and characterization of psEVs, and may exert unavoidable biological effects, which cannot be excluded. Sixth, we investigated the overall functionality of psEVs in emulating a diverse sEV population within human carotid plaques, the respective roles of psEV sub‐populations (e.g., exosomes and small microvesicles) have not been explored. Finally, the composition and function of plEVs were not addressed in this study.

In conclusion, we reported differences in miRNA profiles of psEVs from stable and vulnerable (FCR and IPH) plaques of aCAS patients. Through multistage validation, we identified five plasma‐derived sEV miRNAs as potential noninvasive biomarkers for plaque vulnerability assessment. These plasma‐derived sEV miRNAs, detected by the HCNIB system, hold promise for future community screenings for the risk of vulnerable aCAS. Mechanistically, we demonstrated that miR‐497‐5p mediated the proinflammatory and proatherogenic effects of vulnerable psEVs by directly targeting atheroprotective UCP2. Pharmacological targeting of miR‐497‐5p and UCP2 may represent novel therapeutic approaches to attenuate atherosclerosis development and progression.

## 4 Materials and Methods

A detailed description of materials and methods can be found in the online supplementary data.

### 4.1 Study Design and Participants

We analyzed consecutive patients with aCAS undergoing CEA for severe stenosis (≥70%) at the distal common carotid artery or the extracranial internal carotid artery. This study involved three cohorts: (1) the sequencing cohort, which aimed to phenotypically characterize psEVs, perform miRNA‐seq on psEVs, as well as conduct a correlation analysis of the identified DEmiRNAs between psEVs and plasma‐derived sEVs; (2) the discovery cohort, which aimed to identify potential plasma‐derived sEV miRNAs as indicators of plaque vulnerability, with their psEVs isolated for exploration of biological functions and molecular mechanisms; and (3) the independent external cohort, which aimed to validate the robustness of the identified plasma‐derived sEV miRNAs as diagnostic indicators, with their carotid plaque samples used for the isolation of plaque‐exudative sEVs. The sequencing and discovery cohorts were retrospectively screened from March 1, 2019, to February 20, 2021, from patients admitted to the Department of Neurosurgery, Xuanwu Hospital, Capital Medical University (Beijing, China), using clinical databases and biobanks that were maintained prospectively. The independent external cohort was prospectively recruited from January 1, 2022, to December 31, 2022, from patients admitted to the Department of Stroke Center, Central Hospital affiliated with Shandong First Medical University (Shandong, China). Inclusion criteria were patients with (1) age ≥18 years; and (2) no amaurosis fugax, transient ischemic attack, or ischemic stroke within 6 months prior to CEA. Exclusion criteria were patients with (1) post‐CEA re‐stenosis or radiation‐induced stenosis; (2) acute coronary syndrome or acute peripheral vascular occlusion within the previous 6 months; (3) current malignant diseases; (4) severe liver or kidney failure; and (5) missing clinical data or plaque/blood samples. The degree of carotid artery stenosis was assessed according to the North American Symptomatic Carotid Endarterectomy Trial (NASCET) criteria [68]. Demographic information, medical history, cardiovascular risk factors, medication use, and basic laboratory results were extracted from electronic medical records. Stable, IPH, and FCR plaques were first screened by carotid artery ultrasound, computed tomography angiography, and magnetic resonance imaging [7, 8, 9, 10], and then confirmed by histopathological staining [69, 70, 71]. This study was approved by the Ethics Committees of the Xuanwu Hospital, Capital Medical University (KS2019‐074) and Central Hospital Affiliated to Shandong First Medical University (SZR2021‐006‐01) and performed according to the Principles of Declaration of Helsinki. Written informed consent was obtained from all participants in the study.

## Author Contributions

Xin Xu and Liqun Jiao conceived, designed, and supervised the study. Xin Xu, Liqun Jiao, Taoyuan Lu, and Ran Xu developed the methodology. Yao Feng, Wenbo Cao, Dianwei Liu, Peng Gao, Yan Ma, Yabing Wang, Bin Yang, Yanfei Chen, and Jian Chen performed the recruitment of participants and data collection. Xin Xu, Taoyuan Lu, Yao Feng, Ran Xu, and Xinyu Wang performed experiments. Xin Xu, Taoyuan Lu, Bin Yang, and Yan Ma interpreted the results, performed data analysis, and prepared the figures and tables. Lebin Chen and Yuanyuan Ji provided technical support. Xin Xu wrote the manuscript. Liqun Jiao reviewed and revised the manuscript. All authors provided critical feedback on the manuscript for intellectual content. All authors have read and approved the final manuscript.

## Conflicts of Interest

Authors Lebin Chen and Yuanyuan Ji are employees of Hangzhou Dixiang Co. Ltd. but have no potential relevant financial or nonfinancial interests to disclose. The remaining authors declare no conflicts of interest.

## Ethics Statement

This human study was approved by the Ethics Committees of the Xuanwu Hospital, Capital Medical University (KS2019‐074) and Central Hospital Affiliated to Shandong First Medical University (SZR2021‐006‐01) and was performed according to the Principles of Declaration of Helsinki. Written informed consent was obtained from all participants in this study. The animal study was approved by the Animal Care and Use Committee of Xuanwu Hospital, Capital Medical University (XW‐20210617‐1). All procedures were conducted in strict accordance with the NIH Guide for the Care and Use of Laboratory Animals, and efforts were made to minimize the number of mice used and reduce their suffering.

## Supporting information



Supporting Information

Supporting Information

Supporting Information

## Data Availability

The raw data that support the findings of the current study are available from the corresponding author upon reasonable request.
