# Supplementary material for: Carotid Plaque‐Derived Small Extracellular Vesicles Mediate Atherosclerosis and Correlate With Plaque Vulnerability
Source: MedComm (2020). 2025 May 19;6(6):e70220. doi: 10.1002/mco2.70220 (PMC12086378; doi:10.1002/mco2.70220)
Supplement: Supplementary file 1 — Supporting Information [file MCO2-6-e70220-s003.docx]

**Supplementary data**

**Carotid plaque-derived small extracellular vesicles mediate atherosclerosis and correlate with plaque vulnerability**

Xin Xu^1, 2, 3, *^; Taoyuan Lu^1, 2^; Yao Feng^4^; Wenbo Cao^1, 2^; Dianwei Liu^3, 5^; Peng Gao^1, 2^; Yan Ma^1, 2^; Yabing Wang^1, 2^; Bin Yang^1, 2^; Yanfei Chen^1, 2^; Jian Chen^1, 2^; Ran Xu^1, 2^; Xinyu Wang^1,2^; Lebin Chen^6^, Yuanyuan Ji^6^, Liqun Jiao^1, 2, 3, 7, *^

^1^Department of Neurosurgery, Xuanwu Hospital, Capital Medical University, 45 Changchun Street, Beijing, China

^2^China International Neuroscience Institute (China-INI), 45 Changchun Street, Beijing, China

^3^Xuanwu Jinan Hospital, 5106 Jingshi Road, Jinan, Shandong, China

^4^Department of Neurology, Fuwai Hospital, National Center for Cardiovascular Diseases, Chinese Academy of Medical Sciences and Peking Union Medical College, 167 Beilishi Road, Beijing, China

^5^Department of Stroke Center, Central Hospital Affiliated to Shandong First Medical University, 5106 Jingshi Road, Jinan, Shandong, China

^6^Hangzhou Dixiang Co. Ltd., 279 Zixuan Road, Hangzhou, China

^7^Department of Interventional Neuroradiology, Xuanwu Hospital, Capital Medical University, 45 Changchun Street, Beijing, China

*Co-corresponding authors:

Xin Xu: [xuxindoc@xwhosp.org](mailto:xuxindoc@xwhosp.org) and Liqun Jiao: [jiaoliqun@xwhosp.org](mailto:jiaoliqun@xwhosp.org)

**Materials and methods**

**1. Human carotid plaque collection and processing**

Freshly surgically removed human carotid plaques were immediately processed in the laboratory. The segment with the greatest plaque burden was selected as the culprit lesion and harvested. Tissue slices were obtained from both the cranial and caudal ends of each excised tissue block for histopathological analysis of lesion types, as previously described.[^1-3^](#_ENREF_1) Briefly, intraplaque hemorrhage (IPH) and fibrous cap rupture (FCR) were evaluated by hematoxylin-eosin (H&E; Cat# BSBA-4025; Zsgb-bio, Beijing, China) and Masson (Cat# BP028; Biossci Biotech, Hubei, China) stains. Representative histological images are presented in Figure S9. A stable plaque was defined as the absence of IPH, FCR, or other features of plaque vulnerability based on lipid content, collagen deposition, and macrophage and smooth muscle cells (SMC) burdens. The remaining core of the culprit lesions (IPH, FCR, or stable necrotic core) was snap-frozen in liquid nitrogen, and stored at -80°C for tissue-derived extracellular vesicle (Ti-EV) isolation.

**2 Plaque-derived EV isolation**

Human carotid plEVs and psEVs were isolated following a previously established protocol with some modifications.[^4^](#_ENREF_4) A schematic diagram of the workflow is presented in Figure 1B. Briefly, plaque tissues were weighed (~550 mg), embedded in O.C.T. medium (Tissue-Tek, Torrance, CA), and sliced into 200-μm thick sections using a microtome (Leica Microsystems, Wetzlar, Germany). They were then incubated with a dissociation mixture (Miltenyi Human Tumor Dissociation Kit; Cat# 130-096-929; Miltenyi Biotec, Bergisch Gladbach, Germary) for 15 min at 37°C. The Ti-EV-containing media were filtered through 70-µm MACS® SmartStrainers (Cat# 130-098-462; Miltenyi Biotec), and subsequently subjected to sequential centrifugation at 300g for 10 min, 2,000g for 20 min, and 10,000g for 20 min (SW 32 Ti Swinging-Bucket Rotor; k-factor, 3526.5; acceleration and deceleration at the maximum rate; acceleration time, ~0.8 min; deceleration time, ~2.5 min. Open-Top Thinwall Ultra-Clear Tube with 38.5 ml capacity, Cat# 344058. OptimaTM XPN-100 Ultracentrifuge; Beckman Coulter, Brea, CA, USA) to remove cells and tissue debris. The plEVs were washed and obtained by further centrifugation at 10,000g for 20 min to collect the pellets. The psEV-containing supernatant was filtered through a 0.22-μm syringe filter (Cat# SLGPR33RB, Millipore, Temecula, CA, USA), and then ultracentrifuged at 150,000g for 2 hrs (SW 32 Ti Swinging-Bucket Rotor; k-factor, 238.6; acceleration and deceleration at the maximum rate; acceleration time, ~2.5 min; deceleration time, ~4.2 min. Open-Top Thinwall Ultra-Clear Tube with 38.5 ml capacity. OptimaTM XPN-100 Ultracentrifuge) to obtain the pellets. All centrifugation processes were conducted at 4°C. The isolated psEVs were re-suspended in phosphate buffer solution, purified by SEC using the Exosupur® columns (Cat# Echo9101; Echobiotech, Beijing, China), and concentrated using the Amicon® Ultra Centrifugal Filters (100 kDa; Cat# UFC910008; Millipore). The size distribution and concentration of the obtained Ti-EVs were determined by nanoparticle tracking analysis (NTA) using the NanoSight system (NS300; Malvern Panalytical Inc, Amesbury, UK). The concentration of Ti-EV-derived proteins was measured using a Pierce™ BCA Protein Assay kit (Cat# 23225; ThermoFisher Scientific, Carlsbad, CA, USA). Western blotting was then conducted to detect typical EV markers TSG101, HSP70, and CD63, and a negative marker calnexin. In addition, non-EV contaminants including apolipoprotein A1 (ApoA1; Cat# E-EL-H0125; Elabscience, Hubei, China), ApoB100 (Cat# E-EL-H6171; Elabscience), collagen I (Cat#. ab285250; Abcam, Cambridge, MA, USA), and collagen III (Cat# E-EL-H6049; Elabscience) were measured using commercial enzyme-linked immunosorbent assay (ELISA) kits, following the manufacturer’s instructions.

**3. Plaque-exudative sEV isolation**

Plaque-exudative sEVs were isolated using the explant culture method.[^5^](#_ENREF_5) Briefly, freshly removed human carotid plaques were washed in sterile phosphate-buffered saline (PBS), sliced into 200-μm thick sections, and cultured in DMEM/F12 medium (Gibco, Grand Island, NY, USA) without fetal bovine serum (FBS) at 37°C in a humidified atmosphere with 5% CO_2_ for 12 hrs. Subsequently, the culture medium was harvested, and sEVs released from the plaques were isolated using the same centrifugation, purification, and concentration procedures employed for psEV extraction.

**4. Plasma-derived EV isolation**

Plasma-derived sEVs were isolated from ethylenediaminetetraacetic acid (EDTA)-anticoagulated peripheral venous blood samples collected during hospital admission prior to carotid endarterectomy (CEA). A total of 6 mL of blood samples was subjected to a series of centrifugations: first at 120*g* for 20 min at room temperature (RT), followed by 1,500*g* for 20 min at RT, and finally at 13,000*g* for 20 min at 4°C to yield cell-free plasma. They were then filtered through a 0.22-μm filter (Millipore) and subjected to ultracentrifugation at 150,000*g* for 2 hrs at 4°C, followed by purification through SEC and concentration to obtain plasma-derived sEVs.

**5. Transmission electron microscopy (TEM)**

TEM was performed according to previously established protocols. For TEM observation of plaque tissue,[^6^](#_ENREF_6) plaque tissues were fixed in 4% paraformaldehyde (PFA) and 3% glutaraldehyde for 24 hrs at 4°C, followed by treatment with 1% osmic acid for 2 hrs at 4°C. The samples were then dehydrated in a gradient of ethanol and embedded in araldite. Subsequently, ultrathin sections were cut using an ultramicrotome (Leica EM UC6), stained with 2% uranyl acetate for 4 min and lead citrate for 2 min, and imaged with the Hitachi H-7650 TEM device (Tokyo, Japan). For TEM observation of sEVs,[^7^](#_ENREF_7) the isolated psEVs or plaque-exudative sEVs were fixed with 3% glutaraldehyde overnight at 4°C, subsequently loaded onto glow-discharged 230-mesh formvar/carbon-coated grids (Cat# AZH230; Electron Microscopy China, Beijing, China) for 1 min. They were then negatively stained with 3% uranyl acetate for 1 min, and imaged using the Hitachi H-7650 TEM device.

**6. RNA extraction and quality control**

Total RNA was extracted and purified from psEVs, plaque-exudative sEVs, plasma-derived sEVs, and treated human umbilical cord vein endothelial cells (HUVECs) using the miRNeasy® Mini Kit (Cat# 217004; Qiagen, Hilden, Germany) following the manufacturer’s instructions. RNA concentration and purity were assessed using the Agilent RNA 6000 Nano Kit (Cat# 5067-1511) on an Agilent 2100 Bioanalyzer (Agilent Technologies, Santa Clara, CA, USA).

**7. MicroRNA sequencing (miRNA-seq) of psEVs**

The miRNA-seq of psEVs was performed by a commercial service (Echobiotech, Beijing, China). Briefly, small RNA libraries were constructed using 1ng-10ng of total RNA per sample as input material with the QIAseq miRNA Library Kit (Cat# 331502; Qiagen, Frederick, MD, USA), and each library was indexed using a unique barcode. Reverse transcription primers with unique molecular indices (UMIs) were introduced to analyze the quantification of miRNA expression during complementary DNA (cDNA) synthesis and PCR amplification. Library quality was assessed on an Agilent 2100 Bioanalyzer (Agilent Technologies) using an Agilent High Sensitivity DNA kit (Cat# 5067-4626). The index-coded samples were clustered on a cBot Cluster Generation System (Illumina, San Diego, CA, USA) with TruSeq PE Cluster Kit v3-cBot-HS (Cat# PE-401-3001; Illumina) according to the manufacturer’s instructions. Thereafter, the libraries were sequenced on an Illumina Novaseq 6000 platform, and 150bp paired-end reads were generated. The cleaned reads were obtained after filtering ribosomal RNAs, transfer RNAs, small nuclear RNAs, small nucleolar RNAs, and other non-coding RNAs with Silva, GtRNAdb, Rfam, and Repbase databases by using Bowtie software (version 1.2.1.1).

**8. Bioinformatic analysis of miRNA-seq data**

The Homo sapiens GRCh38 reference genome was utilized for RNA annotations. Known miRNAs were identified by aligning sequences against the miRBase v22 database (http://www.mirbase.org/),[^8^](#_ENREF_8) those unannotated were analyzed using miRDeep2 (version 2.0.5) to identify novel miRNAs.[^9^](#_ENREF_9) The expression matrix of quantified UMI counts for miRNAs was normalized to TPM, with miRNAs exhibiting low expression abundance (average TPM<10) excluded from further analysis. We first identified the DEmiRNAs between stable psEVs (n=5) and vulnerable psEVs (n=13; 5 IPH and 8 FCR), and subsequent subgroup analyses further identified the DEmiRNAs between psEVs derived from vulnerable subtypes IPH (n=5) and FCR (n=8). The cut-oﬀ criteria for DEmiRNAs were uniformly established at log2 fold-change (FC) > 0.58 and *p*-value <0.05, which were identified with two-sided t-tests using the “EdgeR” R package (version 3.12.1).[^10^](#_ENREF_10) Due to the relatively small sample size of the sequencing cohort, we opted not to employ strict p-value correction methods in differential expression analysis to facilitate the identification of more DEmiRNA candidates for diagnostic biomarker exploration. The identified DEmiRNAs were initially classified into up-regulated and down-regulated categories. Subsequently, the target genes for each category were predicted using the TargetScan database (version 8.0; http://www.targetscan.org/), and all predicted miRNA-gene pairs were included in further analysis. GO and KEGG enrichment analyses were performed using the “clusterProfiler” R package (version 4.12.6) to assess the potential functions of the identified target genes, and pathways with a false discovery rate (FDR; Benjamini-Hochberg method) <0.05 were considered significantly enriched. Additionally, the TargetScan database was also utilized to predict the target genes of miR-497-5p, with only validated genes included in subsequent analyses.

**9. Quantitative reverse transcription polymerase chain reaction (qRT-PCR)**

For miRNA analysis, 1 μg of total RNA was reverse transcribed into cDNA using the PrimeScript™ RT reagent Kit (Perfect Real Time; Cat# RR037A; Takara, San Jose, CA, USA). TaqMan™ Advanced miRNA Assays (Cat# A25576; ThermoFisher Scientific) were performed to quantify miRNAs on an Applied Biosystems 7500 Real-Time PCR System (ThermoFisher Scientific). Small nuclear RNA U6 served as the internal reference, and relative fold changes were calculated using the 2^−ΔΔCt^ method. The sequences of primers and probes are listed in Table S10. For UCP mRNA analysis, cDNA was synthesized from 1 μg of total RNA using the SuperScript® III CellsDirect™ cDNA Synthesis Kit (Cat# 18080300; ThermoFisher Scientific). qRT-PCR analysis was performed using the corresponding primers and SYBR Green PCR Master Mix (Cat# 4309155; ThermoFisher Scientific). The mRNA levels of UCP2 (forward 5’- AGATGTGGTAAAGGTCCGCTTC-3’, reverse 5’-GCAATGGTCTTGTAGGCTTCG-3’) were normalized to those of GAPDH (forward 5’-GCCAAGGCTGTGGGCAAGGT-3’, reverse 5’-TCTCCAGGCGGCACGCAGA-3’) using the 2^−ΔΔCt^ method.

**10. High-throughput Nano-bio Chip Integrated System for Liquid Biopsy (HNCIB) and sEV-derived miRNA detection**

The HNCIB system (Figure S5)[^11^](#_ENREF_11) developed by Bixionbiotech Co., Ltd (Hangzhou, China) consists of (1) a 96-well nano-biochip (Bixionbiotech) with immobilized biotin anti-CD63 antibody (Cat# ab134331; Abcam) for capturing and enriching EVs through antigen-antibody interaction; (2) molecular beacons (MBs) containing cationic nanoparticles for miRNA detection. The MBs contain 6-carboxyfluorescein (FAM) dye as the fluorophore and Black Hole Quencher 1 (BHQ1) dye as the quencher; (3) a total internal reflective fluorescence microscopy (TIRFM; Nikon, Tokyo, Japan) system equipped with an Eclipse Ti-E inverted microscope with four laser beams and NIS-Elements AR software (version 4.50.00) for rapid and high-resolution detection of fluorescent signals; and (4) a deep learning algorithm-based data analysis system (Bixionbiotech) for automated image screening and selection, background reduction, and mean fluorescent intensity (MFI) calculation at a single-EV level. To detect sEV-derived miRNAs, isolated psEVs or plasma-derived sEVs (from 90 μL of cell-free plasma samples) were re-suspended in 50 μL of PBS and incubated in the nano-biochip at 37°C for 60 min. After washing, 50 μL of MB-containing cationic nanoparticles in PBS were added to each well, and incubated at 37°C for 60 min. Excess nanoparticles were subsequently removed by rinsing with ultrapure water. Images were captured using the TIRFM system, and data analysis was conducted to obtain MFI. The sequences of MBs for miRNA detection (Sangon Biotech, Shanghai, China) are listed in Table S11.

**11. Cell culture, transfection, and treatments**

HUVECs and human monocytic THP-1 cells were maintained respectively in EGM-2 medium (Allendale, NJ, USA) and RPMI-1640 medium (Gibco) supplemented with 10% FBS and 1% penicillin-streptomycin at 37°C in a humidified atmosphere with 5% CO_2._ The mean protein content of psEVs was measured at 122.29 ± 15.41 μg per plaque. Two stable psEV samples were mixed to form an individual experimental sample, while one sample each of psEVs from IPH and FCR plaques was combined to form a vulnerable psEV experimental sample. Each experiment was conducted in triplicate for each experimental sample. The following three part experiments were conducted:

Part 1: To investigate the impact of psEVs on endothelial inflammation *in vitro*, HUVECs (1×10^5^ cells/well) were seeded in 24-well plates and allowed to reach ~80% confluence. They were then treated with 1 mL of either 1) vehicle PBS, 2) stable psEVs (5 μg/mL), or 3) vulnerable psEVs (5 μg/mL) for 6 hrs.

Part 2: To investigate whether psEVs or non-EV components mediate endothelial inflammation, HUVECs were treated with 1 mL of either 1) vehicle PBS, 2) vulnerable psEVs (5 μg/mL), or 3) Triton X-100 pretreated vulnerable psEVs (5 μg/mL) for 6 hrs. For the lysis of psEVs,[^12^](#_ENREF_12) Triton X-100 (Cat# T8787; Sigma-Aldrich, St. Louis, MO, USA; final concentration of 0.075%, v/v) was mixed with vulnerable psEVs, vortexed for 30 s at RT, and subsequently subjected to ultracentrifugation at 150,000g for 2 hrs at 4 °C to collect the precipitate.

Part 3: (1) We first measured the expression of miR-497-5p in HUVECs treated with different concentrations of oxidized (ox)-LDL (25, 50, 100, and 200 µg/mL; Cat# IO1300; Solarbio) for 24 hrs or 100 µg/mL of ox-LDL for 12, 24, and 48 hrs, respectively; (2) For the functional analysis of miR-497-5p in ox-LDL-treated HUVECs, HUVECs were transfected with either miR-497-5p mimics (100 nM) or their negative control (NC) mimics (100 nM) for 48 hrs, followed by exposure to ox-LDL (100 µg/mL) for 24 hrs; (3) To investigate whether miR-497-5p exerted biological functions by down-regulating uncoupling protein 2 (UCP2), HUVECs were co-transfected with miR-497-5p mimics (100 nM) and UCP2 overexpression lentiviruses (or NC lentiviruses) for 48 hrs followed by exposure to ox-LDL (100 µg/mL) for 24 hrs; and (4) To explore whether vulnerable psEVs promote endothelial inflammation by miR-497-5p delivery and UCP2 suppression, HUVECs were treated with both ox-LDL (100 µg/mL) and vulnerable psEVs (5 μg/mL). Simultaneously, they were transfected with miR-497-5p inhibitors (100 nM), UCP2 overexpression lentiviruses, or their NCs for 48 hrs. The miR-497-5p mimics, miR-497-5p inhibitors, and their corresponding NCs were purchased from Sheweisi Biotech (Tianjin, China). Transient transfection was conducted utilizing Lipofectamine 3000 (Invitrogen, Waltham, MA, USA) following the manufacturer’s recommendations. Lentiviruses containing either UCP2 or the NC were purchased from Genechem (Shanghai, China), and were transfected into HUVECs using HitransG (Genechem) at a multiplicity of infection of 20.

**12. Dual-luciferase reporter assay**

The UCP2 3’untranslated region (3’UTR) with either wild-type (WT) or mutated (MUT) miR-497-5p binding sites, as predicted by TargetScan (http://www.targetscan.org/), was synthesized and inserted into pmirGLO luciferase vectors (Promega, Madison, WI, USA). The constructed vectors (0.5 μg) were subsequently co-transfected into HEK293T cells with miR-497-5p mimics (100 nM) or NC mimics (100 nM), using Lipofectamine 3000 following manufacturer’s instructions (Invitrogen). Luciferase activity was measured 48 hrs post-transfection using the Dual-Luciferase^®^ Reporter Assay System (Cat# E1910; Promega), and was normalized relative to Renilla luciferase activity.

**13. psEV internalization assay**

The psEVs, fluorescently labeled with the membrane-labelling dye PKH26 (Cat# MINI26; Sigma-Aldrich), were incubated with HUVECs in confocal dish at 37°C for 6 hrs. After washout, the cells were fixed in 4% PFA for 10 min at RT, then counterstained with phalloidin (specific targeting F-actin; Cat# CA1620; Solarbio, Beijing, China) and 4′,6-diamidino-2-phenylindole (DAPI; Cat# ab104139; Abcam). The images were acquired using a confocal fluorescence microscope (FluoView 1200; Olympus, Japan).

**14. Hoechst 33342/propidium iodide (PI) staining**

HUVECs subjected to the indicated treatments were vigorously washed with PBS, and subsequently stained using the Hoechst 33342/PI Double Stain Kit (Cat# CA1120; Solarbio) according to the manufacturer’s instructions. Images were captured under a fluorescence microscope (Olympus IX81; Waltham, MA, USA). Positive staining cells from 5 randomly selected fields were quantified using NIH ImageJ software (Version 1.46r, Wayne Rasband, USA).

**15. Monocyte-endothelial cell adhesion assay**

The adherence of THP-1 cells to the treated HUVECs was analyzed as previously described.[^7^](#_ENREF_7) In brief, THP-1 cells were labeled with the fluorescent probe calcein-AM (Cat# C1430; Invitrogen) per the manufacturer’s instructions. The labeled THP-1 cells (5×10^4^ cells/well) were incubated with treated HUVECs in 24-well plates at 37°C for 1 hr. After washout, the adherent THP-1 cells were fixed with 4% PFA, captured using an Olympus IX81 fluorescence microscope, and counted in 5 randomly selected fields of each well using NIH ImageJ software.

**16. Endothelial permeability assay**

Endothelial permeability was evaluated by measuring the leakage of fluorescein isothiocynate (FITC)-conjugated dextran through the monolayer of the treated HUVECs (Cat# ECM644; Millipore, Billerica, MA), as we have previously described.[^7^](#_ENREF_7) Briefly, HUVECs were cultured on the 0.4-µm pore of collagen-coated transwell inserts until conﬂuent, followed by incubation with the indicated treatments for 6 hrs at 37°C. After washout, the cells were incubated with FITC-dextran for 1 hr at 37°C, and the culture media in the bottom chambers were collected to measure MFI (excitation at 485 nm and emission at 535 nm) using a SpectraMax M5 plate-reader (Molecular Devices, Sunnyvale, CA).

**17. Intracellular reactive oxygen species (ROS) production assay**

Intracellular ROS production in the treated HUVECs was measured by ROS-specific probe DCFH-DA (2',7'-Dichlorodihydrofluorescein diacetate) using a commercial kit according to the manufacturer’s instructions (Cat# S0033; Beyotime, Jiangsu, China). The intensity of fluorescence was detected with a SpectraMax M5 plate-reader (excitation at 488 nm and emission at 525 nm; Molecular Devices).

**18. Animals, atherosclerotic model, and treatments**

This animal study was approved by the Animal Care and Use Committee of Xuanwu Hospital, Capital Medical University (XW-20210617-1). All procedures were conducted in strict accordance with the NIH Guide for the Care and Use of Laboratory Animals, and efforts were made to minimize the number of mice used and to reduce their suffering. Male ApoE^–/–^ mice (C57BL/6J background, aged 7 weeks, 18-20 g; Xiao Shu You Tai Biotechnology Co., Ltd, Beijing, China) were housed and bred in a specific pathogen-free facility with 12-hr diurnal cycle. After a week of acclimatization, mice were fed a high-fat diet (D12108C, Research Diets) for 8 weeks to induce atherosclerosis. Concurrent with the high-fat diet, the mice were intravenously injected with 1) vehicle saline (100 μL), 2) stable psEVs (25 μg in 100 μL of saline), or 3) vulnerable psEVs (25 μg in 100 μL of saline) twice per week for 8 weeks (Figure 2G).[^13^](#_ENREF_13) In the animal experiments, four stable psEV samples were mixed to form an individual experimental sample, while two samples each of psEVs from IPH and FCR plaques were combined to form a vulnerable psEV experimental sample. Each experiment was conducted in triplicate for each experimental sample. Twenty-four hours after the last treatment, the mice (aged 16 weeks+24 hrs) were euthanized using 5% isoﬂurane inhalation, followed by cervical dislocation. The entire aorta and carotid arteries were then surgically dissected under a surgical microscope. The carotid artery tissue was exclusively used for western blotting due to its small size, while the aorta tissue was used for IVIS and histopathological analyses.

**19.** **In vivo imaging system (IVIS)**

We utilized an IVIS Lumina II imaging system (PerkinElmer, Waltham, MA, USA) to track the accumulation of psEVs within the atherosclerotic plaque, as described previously.[^7^](#_ENREF_7) Brieﬂy, isolated psEVs, labeled with a lipophilic dye DiR iodide (Cat# 40757ES25; Yeasen, Shanghai, China), were injected intravenously into untreated atherosclerotic ApoE^–/–^ mice. Three hours post-injection, the mice were euthanized to dissect the entire aorta. *Ex vivo* ﬂuorescence images (excitation at 748 nm and emission at 780 nm in the near infrared range) were acquired from the isolated aorta to calculate the total ﬂuorescence intensity (total radiant efficiency).

**20. Atherosclerotic lesion assessment**

For en face analysis,[^14^](#_ENREF_14) the entire aorta was fixed in 4% PFA overnight at 4°C, unfolded longitudinally, and stained using ORO kit (Cat# C0157S; Beyotime). Images were captured with a digital camera (Canon, Tokyo, Japan), and the percentage of lesion area was determined by calculating the total plaque area divided by the total surface area, using NIH ImageJ software. The cross-section of aortic root was stained with H&E.[^14^](#_ENREF_14) Briefly, the extracted aortic roots were cut transversely, fixed in 4% PFA, embedded in paraffin, and cut into 5-μm thick sections. The paraffin sections were then stained with H&E (Zsgb-bio). Images were captured, and the size of atherosclerotic plaque was calculated using NIH ImageJ software.

**21. Western blotting**

Proteins were extracted from purified Ti-EVs, treated HUVECs, or carotid arteries using a commercial kit (Beyotime). As previously described,[^7^](#_ENREF_7) equal amounts of proteins (30 μg per lane) were subjected to sodium dodecyl sulfate-polyacrylamide gel electrophoresis (SDS-PAGE) and subsequently transferred electrophoretically to 0.22-μm polyvinylidene fluoride (PVDF) membranes (Millipore). The membranes were blocked with 5% non-fat milk for 2 hrs at RT, followed by overnight incubation at 4°C with specific primary antibodies against TSG101 (1:1000; Cat# ab125011; Abcam), HSP70 (1:1000; Cat# ab181606; Abcam), CD63 (1:200; Cat# sc-5275; Santa Cruz Biotechnology, Dallas TX, USA), CD9 (1:1000; Cat# ab236630; Abcam), CD81 (1:1000; Cat# ab79559; Abcam), calnexin (1:500; Cat# 10427-2-AP; Proteintech, Rosemont, IL, USA), NLRP3 (1:1000; Cat# 19771-1-AP; Proteintech), CL-caspase-1 (1:1000; Cat# 4199; Cell Signaling Technology, Danvers, MA, USA), GSDMD-NT (1:1000; Cat# 36425; Cell Signaling Technology), IL-1β (1:1000, Cat# ab283822; Abcam), VE-Cadherin (1:1000, Cat# ab33168; Abcam), ICAM-1 (1:1000, Cat# 60299-1-Ig; Proteintech), VCAM-1 (1:1000, Cat# ab134047; Abcam), VEGF-A (1:1000, Cat# ab46154; Abcam), MMP-9 (1:1000, Cat# 24317T; Cell Signaling Technology), UCP2 (1:1000, Cat# 89326S; Cell Signaling Technology), TXNIP (1:1000, Cat# ab188865; Abcam), and GAPDH (1:1000; Cat# 5174; Cell Signaling Technology). After incubating with the species-appropriate horseradish peroxidase (HRP)-conjugated secondary antibodies (1:5000, all from Cell Signaling Technology) for 2 hrs at RT, antibody binding was detected using enhanced chemiluminescence (Pierce; Rockford, IL, USA) under a Bio-Rad Gel Doc Imager (Bio-Rad, Hercules, CA, USA), and quantified via densitometry using NIH ImageJ software.

**22. Statistical analysis**

Statistical and bioinformatics analyses, and visualization were conducted in R (version 4.4.1). Quantitative variables were presented as mean ± standard deviation (SD) or median (inter quartile range; IQR) according to the Shapiro-Wilk’s test. Differences between two groups were analyzed using independent samples t test or Mann-Whitney U test, while differences among three groups were analyzed using the one-way analysis of variance (ANOVA) followed by post hoc Bonferroni's multiple comparison test. Pearson correlation coefficient was used to assess the correlation between miRNA from psEV and plasma-derived sEV miRNAs. To evaluate diagnostic capacities of plasma-derived sEV miRNAs, we conducted ROC analysis and calculated the AUC using the 'pROC' R package (version 1.18.5). Additionally, we compared the AUCs between the discovery cohort and the external cohort using the DeLong method. Statistical significance was set at a two-tailed p<0.05.

**Table S1.** Characterization of large and small EVs isolated from different plaque subtypes

|  | **Stable (n=5)** | **IPH (n=5)** | **FCR (n=8)** | ***p*** |
| --- | --- | --- | --- | --- |
| **Plaque-derived large EVs** |  |  |  |  |
| Size, nm, mean (SD) | 189.72 (24.5) | 190.96 (15.92) | 192 (12.81) | 0.975^a^ |
| Concentration, ×10^10^ particles/100 mg tissue, mean (SD) | 15.72 (3.03) | 16.4 (1.25) | 16.13 (1.39) | 0.857^a^ |
| Protein content, μg/100mg tissue, mean (SD) | 82.32 (13.84) | 81.68 (6.48) | 81.14 (8.98) | 0.978^a^ |
| **Plaque-derived small EVs** |  |  |  |  |
| Size, nm, mean (SD) | 102.74 (7.42) | 105.5 (9.15) | 101.7 (15.28) | 0.858^a^ |
| Concentration, ×10^10^ particles/100 mg tissue, mean (SD) | 9.21 (1.74) | 7.7 (1.18) | 7.31 (2.13) | 0.205^a^ |
| Protein content, μg/100mg tissue, mean (SD) | 23.8 (9.54) | 22.17 (3.97) | 24.49 (5.59) | 0.825^a^ |

Note: ^a^Analysed by one-way ANOVA test; Abbreviations: EV, extracellular vesicles; FCR, fibrous cap rupture; IPH, intraplaque haemorrhage; SD, standard deviation.

**Table S2.** Baseline characteristics of the sequencing cohort (n=18)

| **Characteristic** | **Stable (n=5)** | **IPH (n=5)** | **FCR (n=8)** | ***p*** |
| --- | --- | --- | --- | --- |
| Age, year, median (IQR) | 64 (62-66) | 65 (64-68) | 63.5 (61-67) | 0.835^a^ |
| Gender, male/female, n | 5/0 | 5/0 | 7/1 | 1.000^b^ |
| BMI, kg/m^2^, median (IQR) | 24.3 (24.3-25.5) | 25.7 (25.5-25.8) | 24.6 (24.1-25.3) | 0.418^a^ |
| Hypertension, n (%) | 4 (80) | 4 (80) | 5 (62.5) | 1.000^b^ |
| Diabetes mellitus, n (%) | 1 (20) | 3 (60) | 4 (50) | 0.584^b^ |
| Dyslipidemia, n (%) | 0 (0) | 3 (60) | 7 (87.5) | 0.412^b^ |
| Coronary artery disease, n (%) | 1 (20) | 1 (20) | 1 (12.5) | 1.000^b^ |
| Atrial fibrillation, n (%) | 1 (20) | 0 (0) | 0 (0) | 0.556^b^ |
| Peripheral artery disease, n (%) | 0 (0) | 0 (0) | 1 (12.5) | 1.000^b^ |
| Current smoking, n (%) | 3 (60) | 2 (40) | 3 (37.5) | 0.840^b^ |
| Current drinking, n (%) | 2 (40) | 1 (20) | 3 (37.5) | 1.000^b^ |
| CAS laterality, right/left, n | 2/3 | 4/1 | 4/4 | 0.584^b^ |
| Ipsilateral stenosis, %, median (IQR) | 85.8 (85.3-92.5) | 79.3 (72.8-87.6) | 89.3 (83.5-89.8) | 0.190^a^ |
| Contralateral stenosis >50%, n (%) | 2 (40) | 1 (20) | 1 (12.5) | 0.771^b^ |
| **Admission medications, n (%)** |  |  |  |  |
| Aspirin | 3 (60) | 5 (62.5) | 0 (0) | 0.416^b^ |
| Clopidogrel | 2 (40) | 2 (25) | 3 (60) | 0.604^b^ |
| Statins | 3 (60) | 4 (80) | 4 (50) | 0.824^b^ |
| Anti-hypertensives | 4 (80) | 3 (60) | 4 (50) | 0.824^b^ |
| **Laboratory analysis, median (IQR)** |  |  |  |  |
| Total cholesterol, mmol/L | 2.97 (2.56-3.69) | 3.78 (3.25-3.9) | 2.97 (2.71-3.45) | 0.353^a^ |
| Triglyceride, mmol/L | 1.06 (0.97-1.26) | 1.16 (1-1.82) | 0.78 (0.75-0.92) | 0.136^a^ |
| HDL cholesterol, mmol/L | 0.92 (0.88-1.16) | 0.94 (0.83-1.02) | 0.91 (0.82-1.01) | 0.774^a^ |
| LDL cholesterol, mmol/L | 1.45 (1.41-2.03) | 2.24 (1.81-2.39) | 1.67 (1.35-1.97) | 0.439^a^ |
| Glucose, mmol/L | 4.36 (4.34-4.37) | 5.54 (5.36-6.44) | 5.31 (5-6.01) | 0.128^a^ |

Note: ^a^Analysed by Mann-Whitney U test; ^b^Analysed by Fisher's exact test. Abbreviations: BMI, body mass index; CAS, carotid artery stenosis; FCR, fibrous cap rupture; HDL, high density lipoprotein; IPH, intraplaque haemorrhage; IQR, interquartile range; LDL, low density lipoprotein.

**Table S3.** The candidate differentially expressed microRNAs (DEmiRNAs) identified by miRNA sequencing of psEVs in the sequencing cohort

|  | **Up-regulated**  **(Vulnerable vs. Stable)**  **n=10** | **Down-regulated**  **(Vulnerable vs. Stable)**  **n=11** | **Up-regulated**  **(FCR vs. IPH)**  **n=17** | **Down-regulated**  **(FCR vs. IPH)**  **n=24** |
| --- | --- | --- | --- | --- |
| 1 | hsa-miR-134-5p | hsa-miR-576-5p | hsa-miR-9-3p | hsa-miR-342-5p |
| 2 | hsa-miR-195-5p | hsa-miR-1307-5p | hsa-miR-143-5p | hsa-miR-574-5p |
| 3 | hsa-miR-497-5p | hsa-miR-181a-3p | hsa-miR-133b | unconservative_1_47804 |
| 4 | hsa-miR-152-3p | hsa-miR-625-5p | hsa-miR-133a-3p | hsa-miR-4732-5p |
| 5 | hsa-miR-125b-5p | hsa-miR-204-5p | hsa-miR-23c | hsa-miR-486-3p |
| 6 | hsa-miR-370-3p | hsa-miR-21-5p | hsa-miR-23a-3p | hsa-miR-1255b-5p |
| 7 | hsa-miR-4429 | hsa-miR-199a-5p | hsa-miR-23b-3p | hsa-miR-139-3p |
| 8 | hsa-miR-320c | hsa-miR-32-5p | hsa-miR-30e-5p | hsa-miR-200c-3p |
| 9 | hsa-miR-320d | hsa-miR-203a-3p | hsa-miR-29b-2-5p | hsa-miR-25-5p |
| 10 | hsa-miR-320e | hsa-miR-197-3p | hsa-miR-376c-3p | unconservative_10_393035 |
| 11 |  | hsa-miR-133b | hsa-miR-365a-3p | hsa-miR-1294 |
| 12 |  |  | hsa-miR-365b-3p | hsa-miR-584-5p |
| 13 |  |  | hsa-miR-340-5p | hsa-miR-4433b-5p |
| 14 |  |  | hsa-miR-19b-3p | hsa-miR-505-5p |
| 15 |  |  | hsa-miR-19a-3p | hsa-miR-744-5p |
| 16 |  |  | hsa-miR-101-3p | hsa-miR-423-5p |
| 17 |  |  | hsa-miR-455-5p | hsa-miR-483-5p |
| 18 |  |  |  | hsa-miR-92b-5p |
| 19 |  |  |  | hsa-miR-196a-5p |
| 20 |  |  |  | hsa-miR-196b-5p |
| 21 |  |  |  | hsa-miR-200a-3p |
| 22 |  |  |  | hsa-miR-200b-3p |
| 23 |  |  |  | hsa-miR-203a-3p |
| 24 |  |  |  | hsa-miR-1275 |

Abbreviations: FCR, fibrous cap rupture; IPH, intraplaque haemorrhage.

**Table S5.** Baseline characteristics of the discovery cohort (n=178)

| **Characteristic** | **Total Patients (n=178)** |  | **Subgroup 1** | | |  | **Subgroup 2** | | | |
| --- | --- | --- | --- | --- | --- | --- | --- | --- | --- | --- |
|  |  |  | **Stable (n=64)** | **Vulnerable (IPH+FCR; n=114)** | ***p*** |  | **Stable (n=64)** | **IPH (n=55)** | **FCR (n=59)** | ***p*** |
| Age, year, median (SD) | 65.9 (6.9) |  | 64.5 (7.9) | 66.6 (6.1) | 0.066^a^ |  | 64.5 (7.9) | 66.9 (5.3) | 66.4 (6.9) | 0.148^e^ |
| Gender, male/female, n | 160/18 |  | 55/9 | 105/9 | 0.204^b^ |  | 55/9 | 51/4 | 54/5 | 0.494^b^ |
| BMI, kg/m^2^, median (IQR) | 25.3 (23.6-27) |  | 25.6 (23.7-27.3) | 25 (23.6-27) | 0.667^c^ |  | 25.6 (23.7-27.3) | 25.3 (23.6-26.5) | 25 (23.7-27.1) | 0.898^f^ |
| Hypertension, n (%) | 132 (74.2) |  | 45 (70.3) | 87 (76.3) | 0.484^d^ |  | 45 (70.3) | 44 (80) | 43 (72.9) | 0.467^d^ |
| Diabetes mellitus, n (%) | 73 (41) |  | 23 (35.9) | 50 (43.9) | 0.383^d^ |  | 23 (35.9) | 27 (49.1) | 23 (39) | 0.322^d^ |
| Dyslipidemia, n (%) | 71 (39.9) |  | 26 (40.6) | 45 (39.5) | 1.000^d^ |  | 26 (40.6) | 19 (34.6) | 26 (44.1) | 0.577^d^ |
| Coronary artery disease, n (%) | 48 (27) |  | 17 (26.6) | 31 (27.2) | 1.000^d^ |  | 17 (26.6) | 17 (30.9) | 14 (23.7) | 0.686^d^ |
| Atrial fibrillation, n (%) | 2 (1.1) |  | 1 (1.6) | 1 (0.9) | 1.000^b^ |  | 1 (1.6) | 1 (1.8) | 0 (0) | 0.76^b^ |
| Peripheral artery disease, n (%) | 6 (3.3) |  | 3 (4.7) | 3 (2.6) | 0.668^b^ |  | 3 (4.7) | 1 (1.8) | 2 (3.4) | 0.874^b^ |
| Current smoking, n (%) | 100 (56.2) |  | 38 (59.4) | 62 (54.4) | 0.627^d^ |  | 38 (59.4) | 30 (54.6) | 32 (54.2) | 0.812^d^ |
| Current drinking, n (%) | 65 (36.5) |  | 24 (37.5) | 41 (36) | 0.967^d^ |  | 24 (37.5) | 21 (38.2) | 20 (33.9) | 0.875^d^ |
| CAS laterality, right/left, n | 86/92 |  | 32/32 | 54/60 | 0.856^d^ |  | 32/32 | 23/32 | 31/28 | 0.491^d^ |
| Ipsilateral stenosis, %, median (IQR) | 79.5 (73.6-84.7) |  | 79.9 (73.6-83.6) | 79.3 (73.2-85.3) | 0.796^c^ |  | 79.9 (73.6-83.6) | 82 (74.7-85.7) | 77.4 (72.2-84.4) | 0.228^f^ |
| Contralateral stenosis >50%, n (%) | 33 (18.5) |  | 12 (18.8) | 21 (18.4) | 1.000^d^ |  | 12 (18.8) | 11 (20) | 10 (17) | 0.915^d^ |
| **Admission medications, n (%)** |  |  |  |  |  |  |  |  |  |  |
| Aspirin | 130 (73) |  | 46 (71.9) | 84 (73.7) | 0.932^d^ |  | 46 (71.9) | 44 (80) | 40 (67.8) | 0.329^d^ |
| Clopidogrel | 51 (28.7) |  | 15 (23.4) | 36 (31.6) | 0.327^d^ |  | 15 (23.4) | 18 (32.7) | 18 (30.5) | 0.497^d^ |
| Ticagrelor | 6 (2.9) |  | 3 (4.7) | 3 (2.6) | 0.668^b^ |  | 3 (4.7) | 2 (3.6) | 1 (1.7) | 0.777^b^ |
| Statins | 143 (80.3) |  | 51 (79.7) | 92 (80.7) | 1.000^d^ |  | 51 (79.7) | 43 (78.2) | 49 (83.1) | 0.797^d^ |
| Anti-hypertensives | 130 (73) |  | 43 (67.2) | 87 (76.3) | 0.254^d^ |  | 43 (67.2) | 42 (76.4) | 45 (76.3) | 0.42^d^ |
| **Laboratory analysis, median (IQR)** |  |  |  |  |  |  |  |  |  |  |
| Total cholesterol, mmol/L | 3.33 (2.97-3.86) |  | 3.42 (2.81-4.06) | 3.32 (3.01-3.84) | 0.851^c^ |  | 3.42 (2.81-4.06) | 3.29 (3.04-3.78) | 3.37 (3-3.86) | 0.725^f^ |
| Triglyceride, mmol/L | 1.14 (0.78-1.54) |  | 1.17 (0.8-1.55) | 1.14 (0.78-1.54) | 0.591^c^ |  | 1.17 (0.8-1.55) | 1.02 (0.72-1.48) | 1.21 (0.86-1.56) | 0.425^f^ |
| HDL cholesterol, mmol/L | 1.04 (0.86-1.18) |  | 0.99 (0.86-1.17) | 1.06 (0.88-1.18) | 0.506^c^ |  | 0.99 (0.86-1.17) | 1.08 (0.94-1.15) | 1.04 (0.84-1.21) | 0.793^f^ |
| LDL cholesterol, mmol/L | 1.84 (1.56-2.18) |  | 1.8 (1.45-2.18) | 1.87 (1.59-2.18) | 0.719^c^ |  | 1.8 (1.45-2.18) | 1.81 (1.58-2.04) | 1.97 (1.61-2.25) | 0.463^f^ |
| Glucose, mmol/L | 5.78 (5.05-6.82) |  | 5.78 (4.73-6.79) | 5.8 (5.18-6.88) | 0.394^c^ |  | 5.78 (4.73-6.79) | 6 (5.2-6.88) | 5.72 (5.06-6.82) | 0.624^f^ |

Note: ^a^Analysed by Student's *t* test; ^b^Analysed by Fisher's exact test; ^c^Analysed by Mann-Whitney U test; ^d^Analysed by Chi-square test; ^e^Analysed by ANOVA test; ^f^Analysed by Kruskal-Wallis H test. Abbreviations: BMI, body mass index; CAS, carotid artery stenosis; FCR, fibrous cap rupture; HDL, high density lipoprotein; IPH, intraplaque haemorrhage; IQR, interquartile range; LDL, low density lipoprotein; SD, standard deviation.

**Table S6.** qRT-PCR results for the relative expression levels of the identified DEmiRNAs in plasma-derived sEVs from the discovery cohort (n=178)

|  | **Stable**  **(n=64)** | **Vulnerable**  **(IPH+FCR; n=114)** | ***p***  **(Stable vs. Vulnerable)** | **IPH**  **(n=55)** | **FCR**  **(n=59)** | ***p***  **(IPH vs. FCR)** | ***p***  **(Stable vs. IPH vs. FCR)** |
| --- | --- | --- | --- | --- | --- | --- | --- |
| **Vulnerable vs. Stable**  **(Up-regulated)** | | | | | | | |
| hsa-miR-134-5p | 2.83±0.29 | 3.01±0.91 | 0.059 | 2.96±0.29 | 3.05±1.24 | 0.589 | 0.266 |
| hsa-miR-195-5p | 4.86±0.57 | 4.99±0.87 | 0.215 | 5.01±1.19 | 4.98±0.39 | 0.859 | 0.531 |
| hsa-miR-125b-5p | 4.57±1.24 | 4.72±0.8 | 0.397 | 4.83±0.54 | 4.61±0.97 | 0.134 | 0.31 |
| hsa-miR-370-3p | 5.86±0.92 | 6.07±0.77 | 0.128 | 5.99±1.02 | 6.14±0.42 | 0.313 | 0.175 |
| hsa-miR-4429 | 4.7±1.03 | 5.18±2.21 | 0.05 | 4.86±2.01 | 5.48±2.36 | 0.133 | 0.056 |
| hsa-miR-320c | 1.96±0.49 | 2.06±0.7 | 0.289 | 2.18±0.42 | 1.94±0.88 | 0.063 | 0.082 |
| hsa-miR-320d | 2.89±0.22 | 2.99±0.7 | 0.149 | 3.05±0.67 | 2.94±0.73 | 0.403 | 0.312 |
| hsa-miR-320e | 2.24±0.4 | 2.42±0.82 | 0.052 | 2.3±1.02 | 2.53±0.56 | 0.142 | 0.056 |
| **Vulnerable vs. Stable**  **(Down-regulated)** | | | | | | | |
| hsa-miR-576-5p | 7.2±1.22 | 7.02±1.11 | 0.337 | 7.11±1.26 | 6.94±0.96 | 0.422 | 0.452 |
| hsa-miR-1307-5p | 9.89±0.79 | 9.69±0.62 | 0.078 | 9.63±0.38 | 9.74±0.78 | 0.336 | 0.118 |
| hsa-miR-181a-3p | 9.06±0.48 | 8.98±0.4 | 0.241 | 8.93±0.57 | 9.02±0.11 | 0.252 | 0.255 |
| hsa-miR-625-5p | 7.39±0.83 | 7.15±0.95 | 0.077 | 7.24±0.55 | 7.06±1.21 | 0.304 | 0.135 |
| hsa-miR-21-5p | 5.31±1.27 | 5.09±0.95 | 0.236 | 5.14±0.72 | 5.05±1.13 | 0.611 | 0.398 |
| hsa-miR-199a-5p | 4.8±1.24 | 4.44±1.07 | 0.056 | 4.47±1.05 | 4.42±1.1 | 0.804 | 0.134 |
| hsa-miR-32-5p | 5.97±1.58 | 5.75±0.94 | 0.321 | 5.62±0.39 | 5.88±1.25 | 0.131 | 0.273 |
| hsa-miR-203a-3p | 7.58±1.62 | 7.22±0.84 | 0.099 | 7.37±1.11 | 7.08±0.42 | 0.071 | 0.064 |
| hsa-miR-197-3p | 8.45±2.86 | 8.08±1.39 | 0.33 | 8.14±1.27 | 8.02±1.51 | 0.646 | 0.486 |
| hsa-miR-133b | 2.98±1.13 | 3.17±0.76 | 0.237 | 3.08±0.21 | 3.25±1.04 | 0.222 | 0.258 |
| **FCR vs. IPH**  **(Up-regulated)** | | | | | | | |
| hsa-miR-9-3p | 5.07±1.56 | 5.44±1.51 | 0.124 | 5.22±1.98 | 5.65±0.83 | 0.138 | 0.097 |
| hsa-miR-133b | 2.98±1.13 | 3.17±0.76 | 0.237 | 3.08±0.21 | 3.25±1.04 | 0.222 | 0.258 |
| hsa-miR-133a-3p | 4.13±1.37 | 4.15±0.81 | 0.896 | 4.02±0.78 | 4.28±0.82 | 0.086 | 0.41 |
| hsa-miR-23c | 3.13±0.85 | 3.15±1.16 | 0.891 | 2.98±0.24 | 3.31±1.58 | 0.116 | 0.247 |
| hsa-miR-23b-3p | 2.86±1.02 | 3.13±1.08 | 0.094 | 3.01±0.32 | 3.25±1.47 | 0.224 | 0.125 |
| hsa-miR-30e-5p | 1.92±1.24 | 2.23±1.19 | 0.111 | 2.06±0.56 | 2.38±1.55 | 0.14 | 0.1 |
| hsa-miR-29b-2-5p | 5.41±0.57 | 5.55±1.51 | 0.386 | 5.32±2.06 | 5.76±0.64 | 0.132 | 0.137 |
| hsa-miR-376c-3p | 1.51±1.86 | 1.56±0.72 | 0.844 | 1.48±0.92 | 1.63±0.46 | 0.278 | 0.793 |
| hsa-miR-365a-3p | 4.62±1.39 | 4.9±1.08 | 0.167 | 4.78±0.73 | 5.01±1.33 | 0.251 | 0.2 |
| hsa-miR-365b-3p | 1.07±0.38 | 1.15±0.77 | 0.342 | 1.08±0.65 | 1.22±0.87 | 0.33 | 0.383 |
| hsa-miR-340-5p | 2.2±0.34 | 2.31±1.05 | 0.306 | 2.16±0.53 | 2.45±1.36 | 0.132 | 0.145 |
| hsa-miR-19b-3p | 3.96±0.61 | 4.21±1.24 | 0.068 | 4.08±0.37 | 4.34±1.68 | 0.249 | 0.13 |
| hsa-miR-19a-3p | 4.97±0.97 | 5.16±0.88 | 0.199 | 5.03±0.29 | 5.28±1.19 | 0.12 | 0.146 |
| hsa-miR-101-3p | 2.3±0.59 | 2.51±0.86 | 0.057 | 2.39±0.52 | 2.62±1.08 | 0.146 | 0.066 |
| hsa-miR-455-5p | 5.07±1.56 | 5.44±1.51 | 0.124 | 5.22±1.98 | 5.65±0.83 | 0.138 | 0.097 |
| **FCR vs. IPH**  **(Down-regulated)** | | | | | | | |
| hsa-miR-342-5p | 9.32±2.35 | 9.09±1.13 | 0.463 | 9.23±1.27 | 8.96±0.98 | 0.209 | 0.471 |
| hsa-miR-574-5p | 10.17±2.04 | 9.56±2.68 | 0.09 | 9.86±1.53 | 9.28±3.42 | 0.24 | 0.134 |
| unconservative_1_47804 | 5.51±0.63 | 5.2±1.62 | 0.068 | 5.31±0.72 | 5.09±2.14 | 0.457 | 0.229 |
| hsa-miR-4732-5p | 5.46±1.18 | 5.13±1.49 | 0.105 | 5.3±0.96 | 4.97±1.85 | 0.23 | 0.142 |
| hsa-miR-486-3p | 8.06±1.49 | 7.7±2.58 | 0.238 | 8.03±2.85 | 7.39±2.28 | 0.19 | 0.187 |
| hsa-miR-1255b-5p | 6.45±0.63 | 6.24±0.98 | 0.078 | 6.36±0.32 | 6.12±1.32 | 0.178 | 0.099 |
| hsa-miR-139-3p | 4.49±1.23 | 4.17±1.34 | 0.108 | 4.34±0.56 | 4.01±1.78 | 0.178 | 0.118 |
| hsa-miR-200c-3p | 10.28±2.98 | 9.73±2.11 | 0.193 | 9.91±1.32 | 9.56±2.64 | 0.368 | 0.27 |
| hsa-miR-25-5p | 7.81±1.74 | 7.39±1.23 | 0.089 | 7.57±1.05 | 7.22±1.37 | 0.127 | 0.076 |
| unconservative_10_393035 | 5.18±0.47 | 5.02±2.03 | 0.413 | 5.11±1.87 | 4.93±2.18 | 0.636 | 0.693 |
| hsa-miR-1294 | 6.21±0.39 | 6.02±0.95 | 0.06 | 5.96±1.19 | 6.07±0.67 | 0.548 | 0.235 |
| hsa-miR-584-5p | 9.75±1.02 | 9.52±0.69 | 0.111 | 9.63±0.48 | 9.42±0.83 | 0.098 | 0.083 |
| hsa-miR-4433b-5p | 10.93±3.23 | 10.41±2.85 | 0.281 | 10.64±2.47 | 10.19±3.17 | 0.398 | 0.39 |
| hsa-miR-505-5p | 7.9±0.61 | 7.71±0.81 | 0.072 | 7.83±0.95 | 7.59±0.63 | 0.117 | 0.056 |
| hsa-miR-744-5p | 9.85±2.34 | 9.57±1.1 | 0.371 | 9.67±1.14 | 9.48±1.07 | 0.362 | 0.468 |
| hsa-miR-423-5p | 5.34±1.21 | 5.04±1.39 | 0.136 | 5.15±0.65 | 4.94±1.83 | 0.41 | 0.252 |
| hsa-miR-483-5p | 10.78±3.02 | 10.17±3.17 | 0.206 | 10.59±3.56 | 9.78±2.74 | 0.178 | 0.177 |
| hsa-miR-92b-5p | 5.14±0.72 | 4.76±1.87 | 0.057 | 4.98±1.19 | 4.56±2.32 | 0.222 | 0.108 |
| hsa-miR-196a-5p | 5.31±1.46 | 4.99±1.38 | 0.157 | 5.08±1.06 | 4.91±1.63 | 0.508 | 0.29 |
| hsa-miR-196b-5p | 9.72±1.33 | 9.37±1.65 | 0.125 | 9.51±1.45 | 9.24±1.82 | 0.381 | 0.229 |
| hsa-miR-200a-3p | 7.62±2.28 | 7.21±1.37 | 0.193 | 7.46±0.52 | 6.98±1.81 | 0.053 | 0.114 |
| hsa-miR-200b-3p | 8.51±0.74 | 8.29±1.7 | 0.224 | 8.42±2.29 | 8.16±0.84 | 0.429 | 0.38 |
| hsa-miR-203a-3p | 7.58±1.62 | 7.22±0.84 | 0.099 | 7.37±1.11 | 7.08±0.42 | 0.071 | 0.064 |
| hsa-miR-1275 | 8.36±0.87 | 8.16±0.9 | 0.139 | 8.29±0.57 | 8.03±1.12 | 0.117 | 0.102 |

Note: Data were presented as the mean ± SD, and were analyzed by t-test or one-way ANOVA. Abbreviations: FCR, fibrous cap rupture; IPH, intraplaque haemorrhage.

**Table S7.** Baseline characteristics of the external cohort (n=82)

| **Characteristic** | **Total Patients (n=82)** |  | **Subgroup 1** | | |  | **Subgroup 2** | | | |
| --- | --- | --- | --- | --- | --- | --- | --- | --- | --- | --- |
|  |  |  | **Stable (n=42)** | **Vulnerable (IPH+FCR; n=40)** | ***p*** |  | **Stable (n=42)** | **IPH (n=18)** | **FCR (n=22)** | ***p*** |
| Age, year, median (SD) | 65.4 (7.6) |  | 64.8 (7.9) | 66.1 (7.4) | 0.456^a^ |  | 64.8 (7.9) | 67.7 (6.4) | 64.7 (7.9) | 0.285^e^ |
| Gender, male/female, n | 74/8 |  | 36/6 | 38/2 | 0.265^b^ |  | 36/6 | 18/0 | 20/2 | 0.292^b^ |
| BMI, kg/m^2^, median (SD) | 25.2 (3.1) |  | 25.5 (3.4) | 24.9 (2.7) | 0.4^a^ |  | 25.5 (3.4) | 24.5 (2) | 25.2 (3.2) | 0.374^e^ |
| Hypertension, n (%) | 53 (64.6) |  | 28 (66.7) | 25 (62.5) | 0.87^c^ |  | 28 (66.7) | 13 (72.2) | 12 (54.6) | 0.47^c^ |
| Diabetes mellitus, n (%) | 36 (43.9) |  | 16 (38.1) | 20 (50) | 0.388^c^ |  | 16 (38.1) | 7 (38.9) | 13 (59.1) | 0.244^c^ |
| Dyslipidemia, n (%) | 17 (20.7) |  | 11 (26.2) | 6 (15) | 0.329^c^ |  | 11 (26.2) | 2 (11.1) | 4 (18.2) | 0.421^b^ |
| Coronary artery disease, n (%) | 25 (30.5) |  | 14 (33.3) | 11 (27.5) | 0.739^c^ |  | 14 (33.3) | 6 (33.3) | 5 (22.7) | 0.652^c^ |
| Atrial fibrillation, n (%) | 1 (1.2) |  | 0 (0) | 1 (2.5) | 0.488^b^ |  | 0 (0) | 0 (0) | 1 (4.55) | 0.488^b^ |
| Peripheral artery disease, n (%) | 0 (0) |  | 0 (0) | 0 (0) | - |  | 0 (0) | 0 (0) | 0 (0) | - |
| Current smoking, n (%) | 56 (68.3) |  | 29 (69.1) | 27 (67.5) | 1^c^ |  | 29 (69.1) | 11 (61.1) | 16 (72.7) | 0.726^c^ |
| Current drinking, n (%) | 48 (58.5) |  | 23 (54.8) | 25 (62.5) | 0.626^c^ |  | 23 (54.8) | 13 (72.2) | 12 (54.6) | 0.411^c^ |
| CAS laterality, right/left, n | 40/42 |  | 19/23 | 21/19 | 0.662^c^ |  | 19/23 | 12/6 | 9/13 | 0.216^c^ |
| Ipsilateral stenosis, %, median (SD) | 78.7 (6.5) |  | 77 (6.3) | 80.5 (6.3) | 0.014^a^ |  | 77 (6.3) | 80.2 (6.8) | 80.7 (5.9) | 0.054^e^ |
| Contralateral stenosis >50%, n (%) | 26 (31.7) |  | 12 (28.6) | 14 (35) | 0.698^c^ |  | 12 (28.6) | 7 (38.9) | 7 (31.8) | 0.734^c^ |
| **Admission medications, n (%)** |  |  |  |  |  |  |  |  |  |  |
| Aspirin | 60 (73.2) |  | 29 (69.1) | 31 (77.5) | 0.539^c^ |  | 29 (69.1) | 14 (77.8) | 17 (77.3) | 0.688^c^ |
| Clopidogrel | 30 (36.6) |  | 19 (45.2) | 11 (27.5) | 0.151^c^ |  | 19 (45.2) | 4 (22.2) | 7 (31.8) | 0.205^c^ |
| Ticagrelor | 1 (1.2) |  | 1 (2.4) | 0 (0) | 1^b^ |  | 1 (2.4) | 0 (0) | 0 (0) | 1^b^ |
| Statins | 67 (81.7) |  | 37 (88.1) | 30 (75) | 0.212^c^ |  | 37 (88.1) | 15 (83.3) | 15 (68.2) | 0.144^c^ |
| Anti-hypertensives | 48 (58.5) |  | 25 (59.5) | 23 (57.5) | 1^c^ |  | 25 (59.5) | 13 (72.2) | 10 (45.5) | 0.228^c^ |
| **Laboratory analysis, median (IQR)** |  |  |  |  |  |  |  |  |  |  |
| Total cholesterol, mmol/L | 3.53 (3.08-4.16) |  | 3.28 (2.98-3.92) | 3.67 (3.12-4.31) | 0.11^d^ |  | 3.28 (2.98-3.92) | 3.41 (3.11-4.11) | 3.91 (3.39-4.41) | 0.123^f^ |
| Triglyceride, mmol/L | 1.17 (0.86-1.55) |  | 1.1 (0.81-1.31) | 1.33 (0.92-1.9) | 0.019^d^ |  | 1.1 (0.81-1.31) | 1.33 (1.04-1.78) | 1.31 (0.89-1.95) | 0.05^f^ |
| HDL cholesterol, mmol/L | 1.12 (0.96-1.35) |  | 1.18 (0.97-1.4) | 1.03 (0.96-1.21) | 0.106^d^ |  | 1.18 (0.97-1.4) | 1.06 (0.96-1.17) | 1.02 (0.9-1.23) | 0.266^f^ |
| LDL cholesterol, mmol/L | 1.77 (1.49-2.44) |  | 1.71 (1.4-2.11) | 1.96 (1.59-2.7) | 0.038^d^ |  | 1.71 (1.4-2.11) | 1.64 (1.54-2.23) | 2.28 (1.7-2.71) | 0.03^f^ |
| Glucose, mmol/L | 5.7 (5-7) |  | 5.5 (5.05-6.81) | 5.8 (4.97-7.23) | 0.358^d^ |  | 5.5 (5.05-6.81) | 5.86 (5.18-7.9) | 5.8 (4.88-6.84) | 0.553^f^ |

Note: ^a^Analysed by Student's *t* test; ^b^Analysed by Fisher's exact test; ^c^Analysed by Chi-square test; ^d^Analysed by Mann-Whitney U test; ^e^Analysed by ANOVA test; ^f^Analysed by Kruskal-Wallis H test. Abbreviations: BMI, body mass index; CAS, carotid artery stenosis; FCR, fibrous cap rupture; HDL, high density lipoprotein; IPH, intraplaque haemorrhage; IQR, interquartile range; LDL, low density lipoprotein; SD, standard deviation.

**Table S8.** Comparison of the area under the curve (AUC) values between the discovery cohort and the independent external cohort using the DeLong method

|  | **Discovery cohort (n=178)** | **External cohort (n=82)** | ***p*** |
| --- | --- | --- | --- |
| **Stable vs. Vulnerable** | | | |
| miR-497-5p | 0.819 (95%CI, 0.757-0.881) | 0.888 (95%CI, 0.814-0.963) | 0.406 |
| miR-152-3p | 0.796 (95%CI, 0.731-0.86) | 0.841 (95%CI, 0.756-0.926) | 0.717 |
| miR-204-5p | 0.898 (95%CI, 0.847-0.948) | 0.913 (95%CI, 0.847-0.980) | 0.164 |
| Combined | 0.981 (95%CI, 0.968-0.995) | 0.993 (95%CI, 0.983-1.000) | 0.197 |
| **IPH vs. FCR** | | | |
| mR-23a-3p | 0.823 (95%CI, 0.746-0.899) | 0.702 (95%CI, 0.537-0.867) | 0.198 |
| miR-143-5p | 0.802 (95%CI, 0.722-0.883) | 0.692 (95%CI, 0.526-0.858) | 0.247 |
| Combined | 0.89 (95%CI, 0.832-0.948) | 0.795 (95%CI, 0.658-0.933) | 0.221 |
| **IPH vs. other (Stable + FCR)** | | | |
| miR-23a-3p | 0.821 (95%CI, 0.759-0.883) | 0.734 (95%CI, 0.598-0.869) | 0.252 |
| **FCR vs. other (Stable + IPH)** | | | |
| miR-143-5p | 0.846 (95%CI, 0.785-0.907) | 0.73 (95%CI, 0.595-0.864) | 0.124 |

Note: Data were presented as the AUC (95% confidence interval), and were analyzed by DeLong's test. Abbreviations: CI, confidence interval; FCR, fibrous cap rupture; IPH, intraplaque haemorrhage.

**Table S10.** Sequences of primers and probes in qRT-PCR

| **Name** | **Sequences** |
| --- | --- |
| miR152-3p (Forward) | 5’-GGCTCAGTGCATGACAGA-3’ |
| miR152-3p (Reverse) | 5’-TCGTATCCAGTGCAGGGTCCGAGGTATTCGCACTGGATACGACCCAAGT-3’ |
| miR152-3p (Probe) | 5’-TTCGCACTGGATACGACCCAAGT-3’ |
| miR204-5p (Forward) | 5’-AGGCGTTCCCTTTGTCATC-3’ |
| miR204-5p (Reverse) | 5’-GTCGTATCCAGTGCAGGGTCCGAGGTATTCGCACTGGATACGACAGGCAT-3’ |
| miR204-5p (Probe) | 5’-TTCGCACTGGATACGACAGGCAT-3’ |
| miR497-5p (Forward) | 5’-ACCGCAGCAGCACACT-3’ |
| miR497-5p (Reverse) | 5’-GTCGTATCCAGTGCAGGGTCCGAGGTATTCGCACTGGATACGACACAAAC-3’ |
| miR497-5p (Probe) | 5’-TTCGCACTGGATACGACACAAACCA-3’ |
| miR-23-3p (Forward) | 5’-CCGCATCACATTGCCAGG-3’ |
| miR-23-3p (Reverse) | 5’-GTCGTATCCAGTGCAGGGTCCGAGGTATTCGCACTGGATACGACGGAAATC-3’ |
| miR-23-3p (Probe) | 5’-TCGCACTGGATACGACGGAAATC-3’ |
| miR143-5p (Forward) | 5’-AGGTGCAGTGCTGCATC-3’ |
| miR143-5p (Reverse) | 5’-GTCGTATCCAGTGCAGGGTCCGAGGTATTCGCACTGGATACGACACCAGA-3’ |
| miR143-5p (Probe) | 5’-TTCGCACTGGATACGACACCAGA-3’ |

**Table S11.** Sequences of molecular beacons for miRNA detection by High-throughput Nano-bio Chip Integrated System for Liquid Biopsy (HNCIB)

| **Name** | **Sequences** |
| --- | --- |
| miR152-3p-G6MB | 5’-6FAM-CCAAGTTCTGTCATGCACTGACTTG-BHQ1-3’ |
| miR204-5p-G6MB | 5’-6FAM-AGGCATAGGATGACAAAGGGAAATGCC-BHQ1-3’ |
| miR497-5p-G6MB | 5’-6FAM-ACAAACCACAGTGTGCTGCTGG-BHQ1-3’ |
| miR-23-3p-G6MB | 5’-6FAM-TGGAAATCCCTGGCAATGTGATATTTCC-BHQ1-3’ |
| miR143-5p-G6MB | 5’-6FAM-CCGCGACCAGAGATGCAGCACTGCACCCGCG-BHQ1-3’ |

Note: Molecular beacons (MBs) containing cationic nanoparticles for miRNA detection. The MBs contain 6-carboxyfluorescein (FAM) dye as the fluorophore and Black Hole Quencher 1 (BHQ1) dye as the quencher.

**Supplementary Figures**


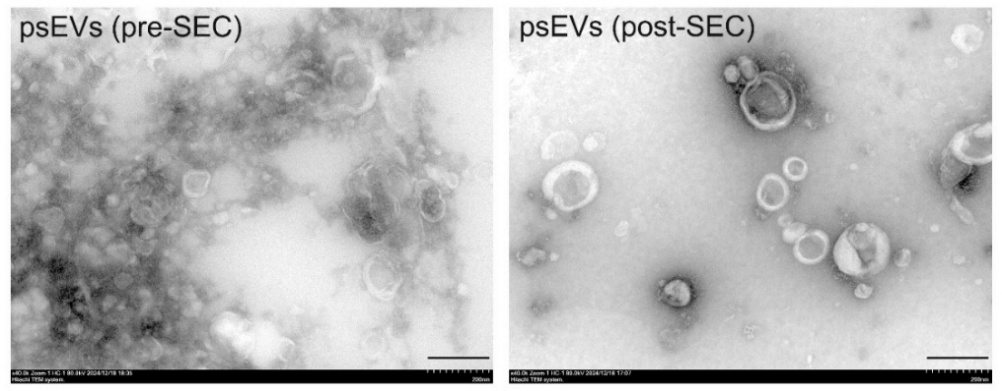


**Figure S1** Representative TEM images of the isolated psEVs pre- and post-SEC. Scale bar = 200 nm.


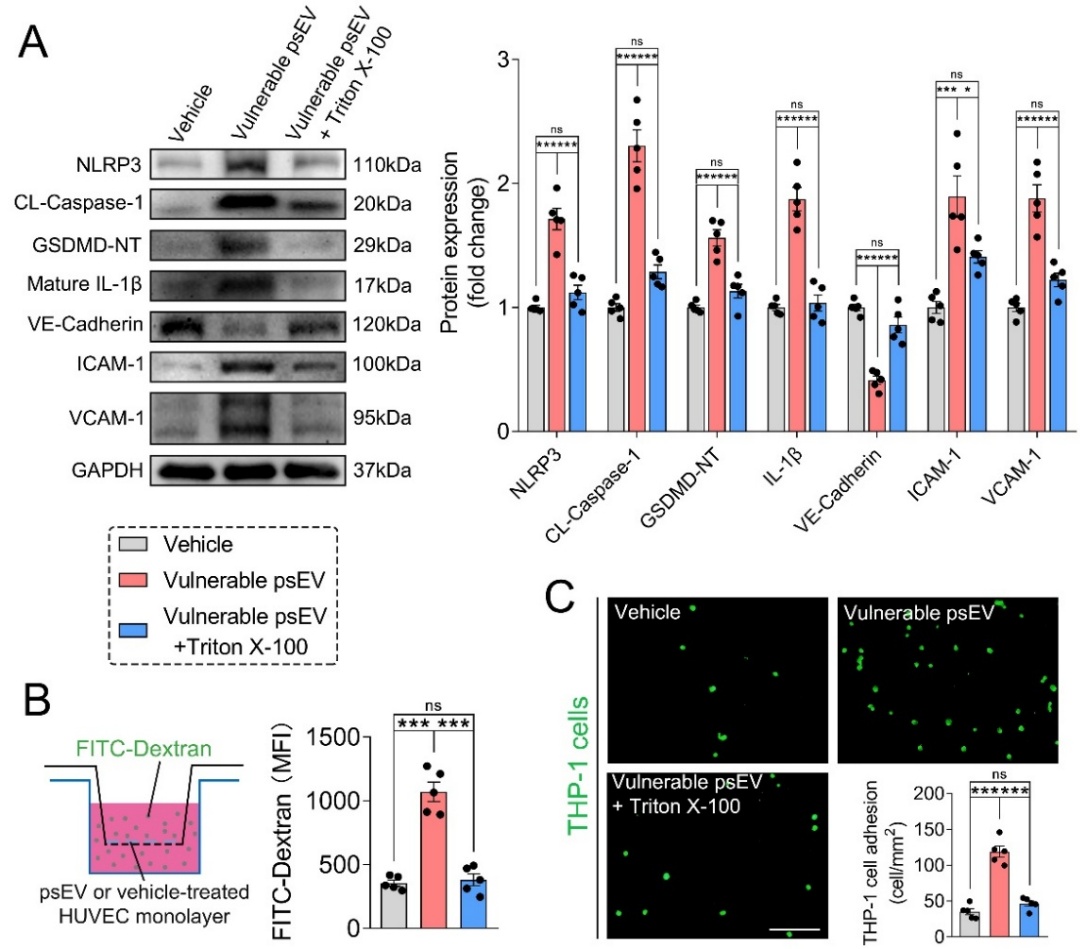


**Figure S2** Triton X-100 pretreatment abrogated vulnerable psEV-induced endothelial inflammation *in vitro*. **A** Representative western blotting bands and densitometric quantifications of NLRP3, cleaved-caspase-1, N-terminal GSDMD, mature IL-1β, VE-cadherin, ICAM-1, and ICAM-1 in the treated HUVECs (n = 5/group). **B** Endothelial permeability measured by FITC-dextran (70 kDa) trans-endothelial leakage (n = 5/group). **C** Representative microphotographs and quantitative analysis of the adhesion of THP-1 monocytes to treated HUVECs (n = 5/group). Scale bar = 200 μm. Technical replicates = 3. Data were presented as the mean ± SD, and were analyzed by one-way ANOVA followed by Bonferroni's multiple comparison test. *p < 0.05 and ***p < 0.001. ns, not significant.


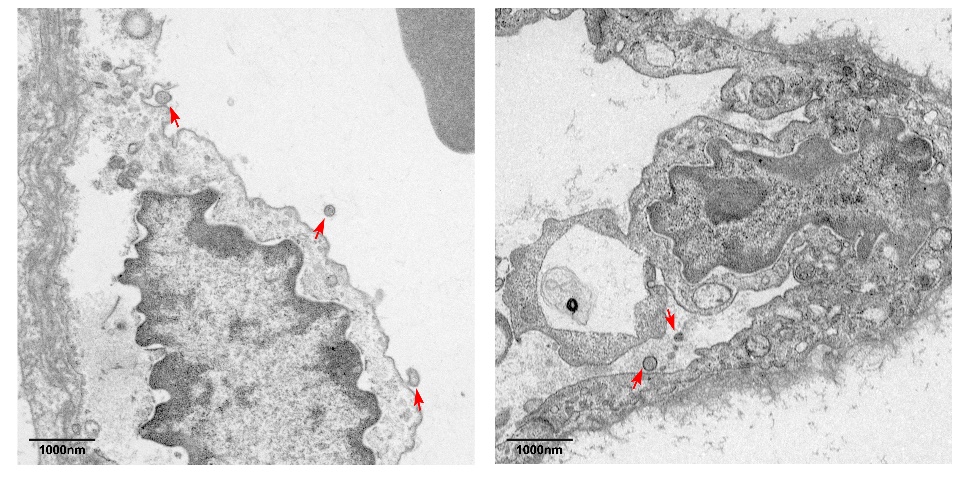


**Figure S3** Representative TEM images of plaque ultrathin sections from atherosclerotic carotid plaques in apoE^-/-^ mice showed the presence of vesicles in the extracellular spaces (red arrows). Scale bar = 1000 nm.


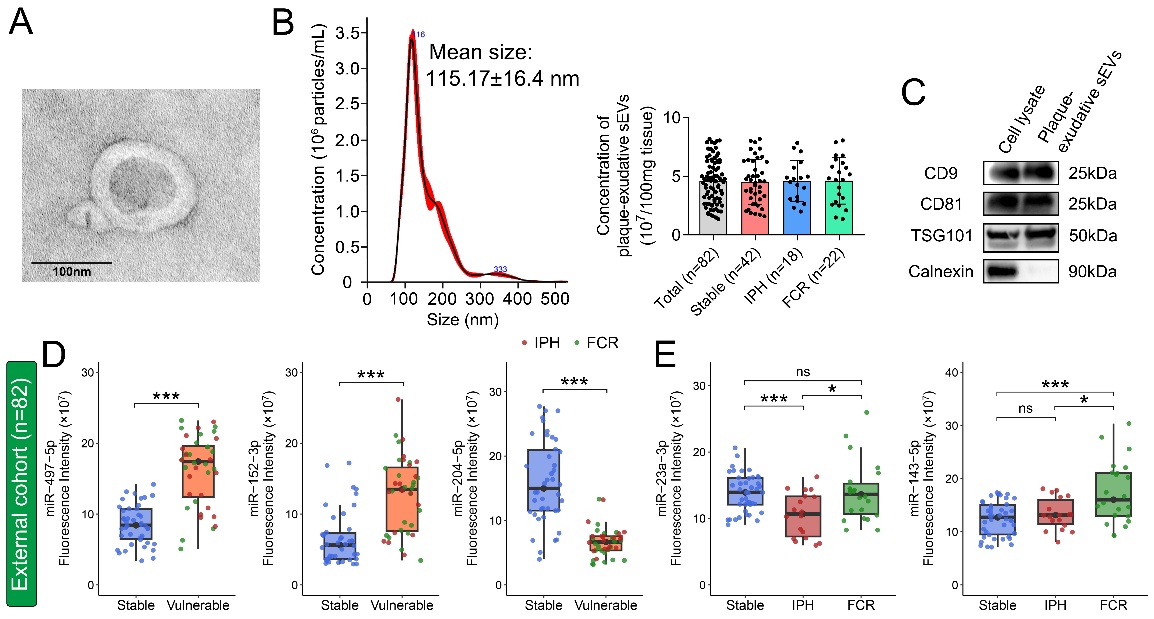


**Figure S4 A-B** Characterization of the isolated carotid plaque-exudative sEVs from aCAS patients in the external cohort (n=82). **A** Representative TEM images of the isolated plaque-exudative sEVs. Scale bar = 100 nm. **B** Size distribution of the isolated plaque-exudative sEVs detected by NTA, and the particle concentration were obtained. **C** Representative western blotting bands of typical EV markers CD9, CD81, and TSG101, and negative EV marker calnexin. **D-E** The relative expression levels of plaque-exudative sEV miR-497-5p, miR-152-3p, miR-204-5p, miR-23a-3p, and miR-143-5p in aCAS patients with stable (blue) or vulnerable (IPH: red; FCR: green) plaques in the external cohort (n = 82). Technical replicates = 3. Data were presented as the mean ± SD, and were analyzed by t-test or one-way ANOVA followed by Bonferroni's multiple comparison test. *p < 0.05 and ***p < 0.001. ns: not significant.


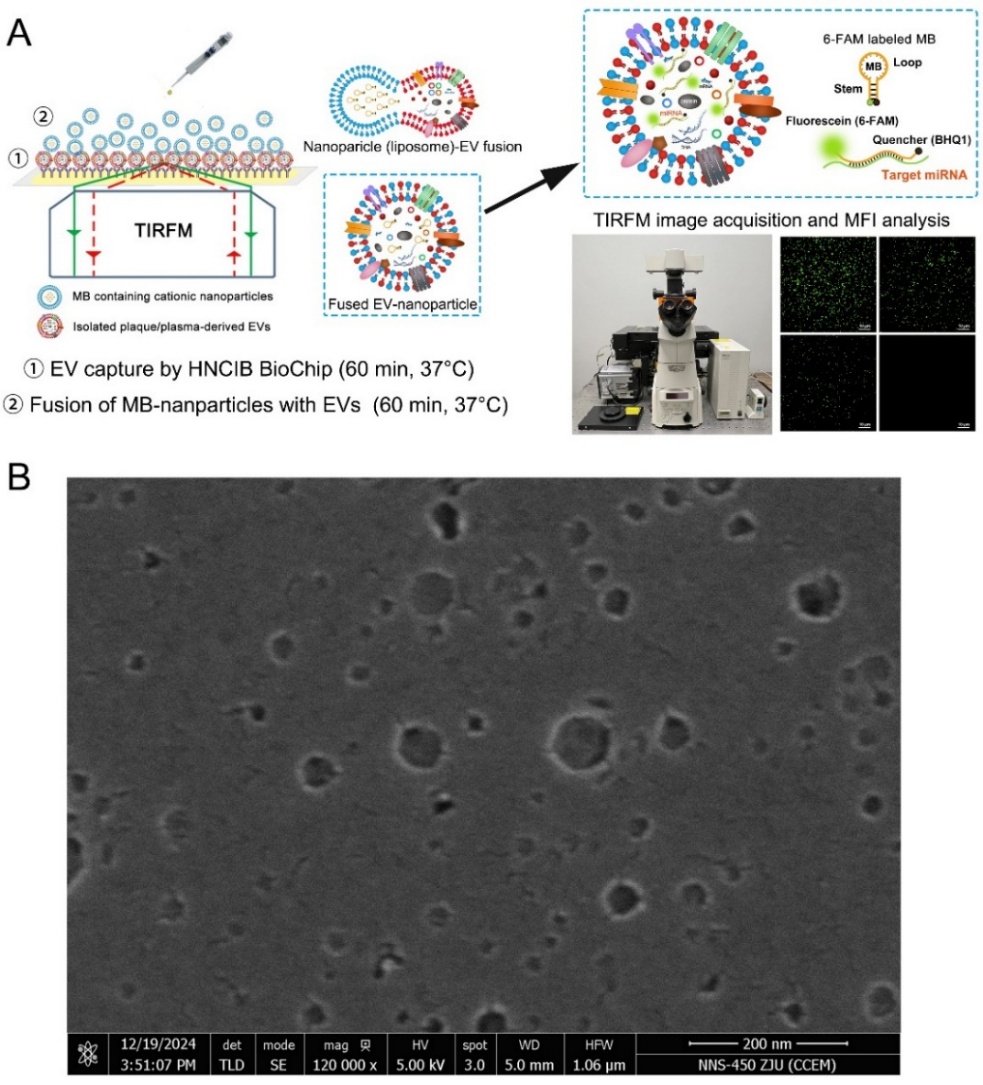


**Figure S5** **A** The illustration of HNCIB system for miRNA detection at a single-EV level. **B** psEVs were captured using a nano-biochip and subsequently detected through TEM imaging. The average particle density was ~10^6^ particles/mm^2^, which was an order of magnitude higher than that achieved by other technologies.[^11^](#_ENREF_11) Scale bar = 200 nm.


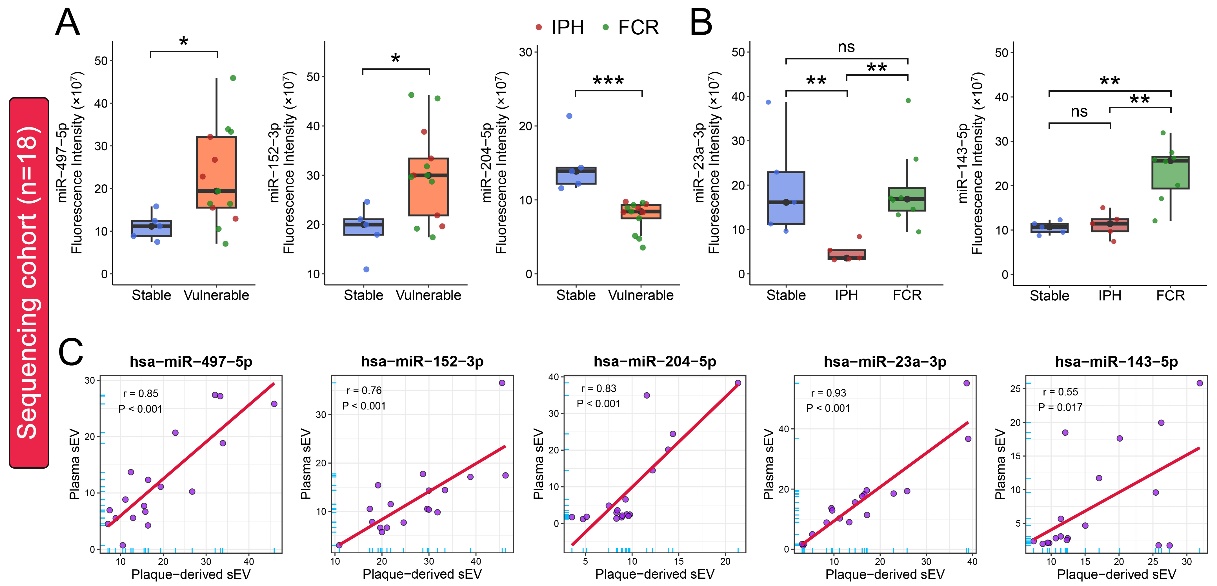


**Figure S6 A-B** The relative expression levels of plasma-derived sEV miR-497-5p, miR-152-3p, miR-204-5p, miR-23a-3p, and miR-143-5p detected by HNCIB system in aCAS patients with stable (blue; n = 5) or vulnerable (IPH: red, n = 5; FCR: green, n = 8) plaques in the sequencing cohort. Technical replicates = 3. Data were presented as the mean ± SD, and were analyzed by t-test or one-way ANOVA followed by Bonferroni's multiple comparison test. *p < 0.05, **p < 0.01, and ***p < 0.001. ns: not significant. **C** Pearson correlation coefficient analyses of correlations between DEmiRNAs from psEVs and plasma-derived sEVs in the sequence cohort (n = 18).


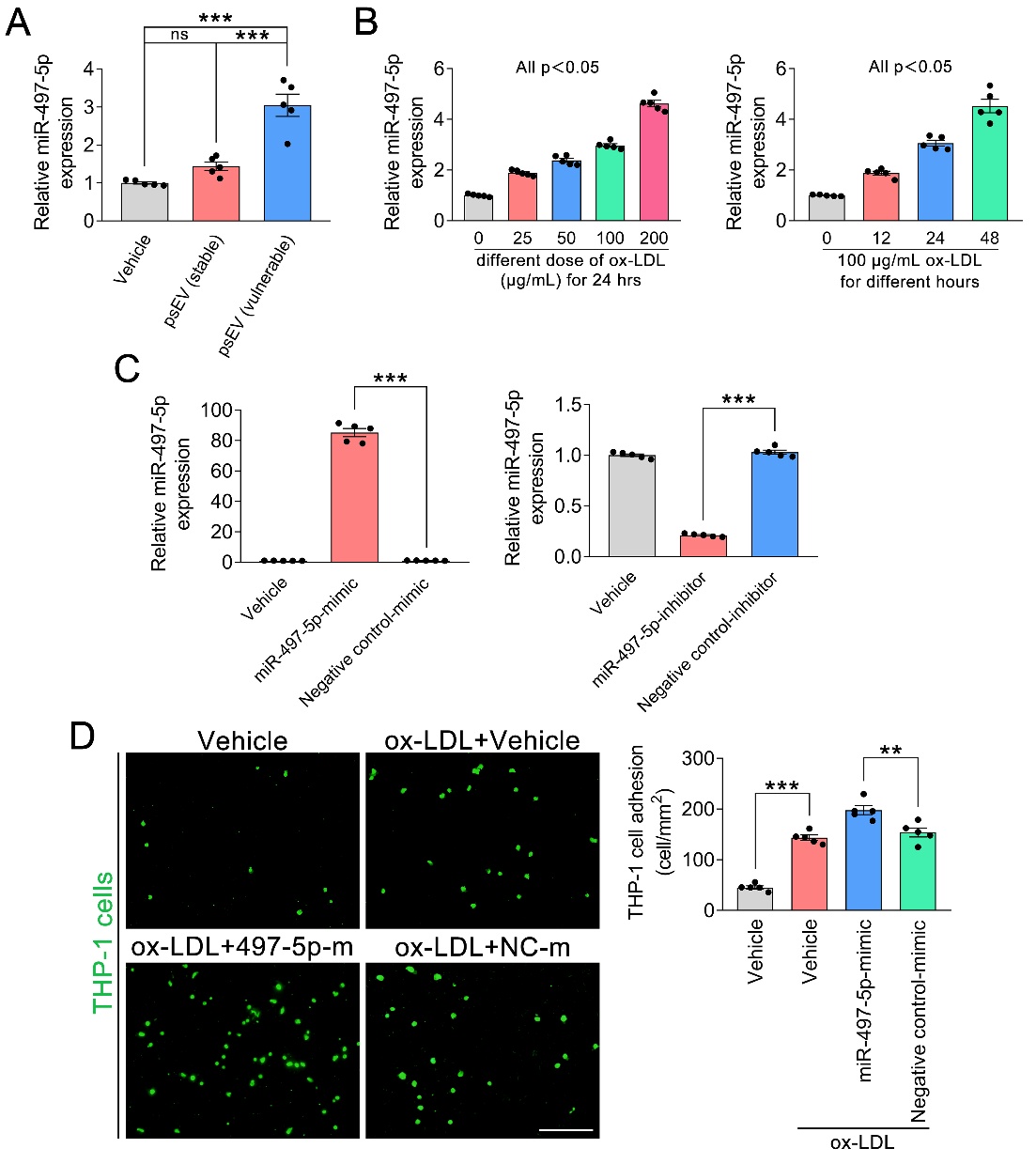


**Figure S7** **A-C** The relative expression of miR-497-5p in the treated HUVECs detected by qRT-PCR (n = 5/group). **D** Representative microphotographs and quantitative analysis of the adhesion of THP-1 monocytes to the treated HUVECs (n = 5/group). Scale bar = 200 μm. Technical replicates = 3. Data were presented as the mean ± SD, and were analyzed by one-way ANOVA followed by Bonferroni's multiple comparison test. **p < 0.01 and ***p < 0.001. ns: not significant.


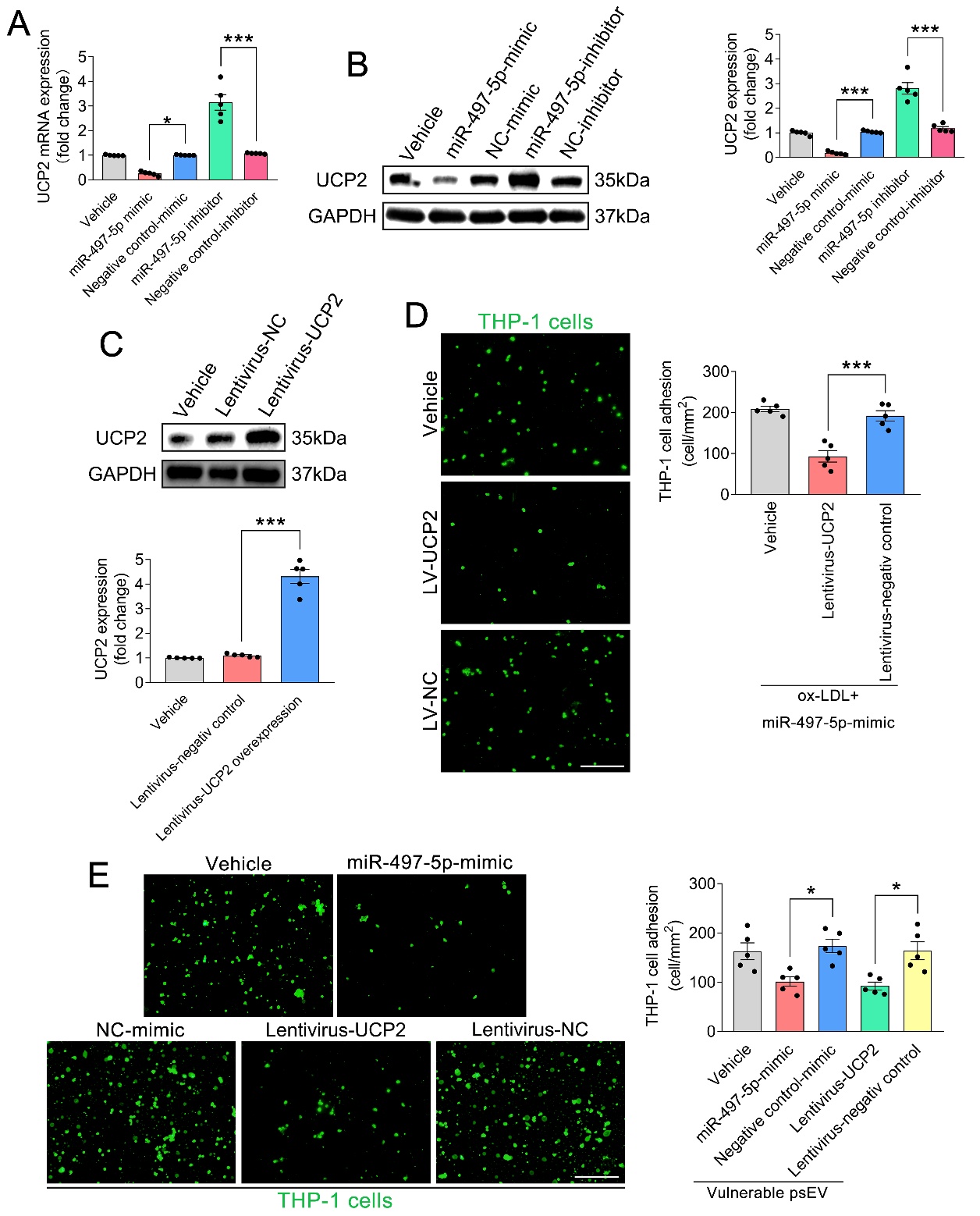


**Figure S8** **A** The relative expression of UCP2 mRNA in the treated HUVECs detected by qRT-PCR (n = 5/group). **B-C** Representative western blotting bands and densitometric quantifications of UCP2 in the treated HUVECs (n = 5/group). **D-E** Representative microphotographs and quantitative analysis of the adhesion of THP-1 monocytes to the treated HUVECs (n = 5/group). Scale bar = 200 μm. Technical replicates = 3. Data were presented as the mean ± SD, and were analyzed by one-way ANOVA followed by Bonferroni's multiple comparison test. *p < 0.05 and ***p < 0.001. ns: not significant.


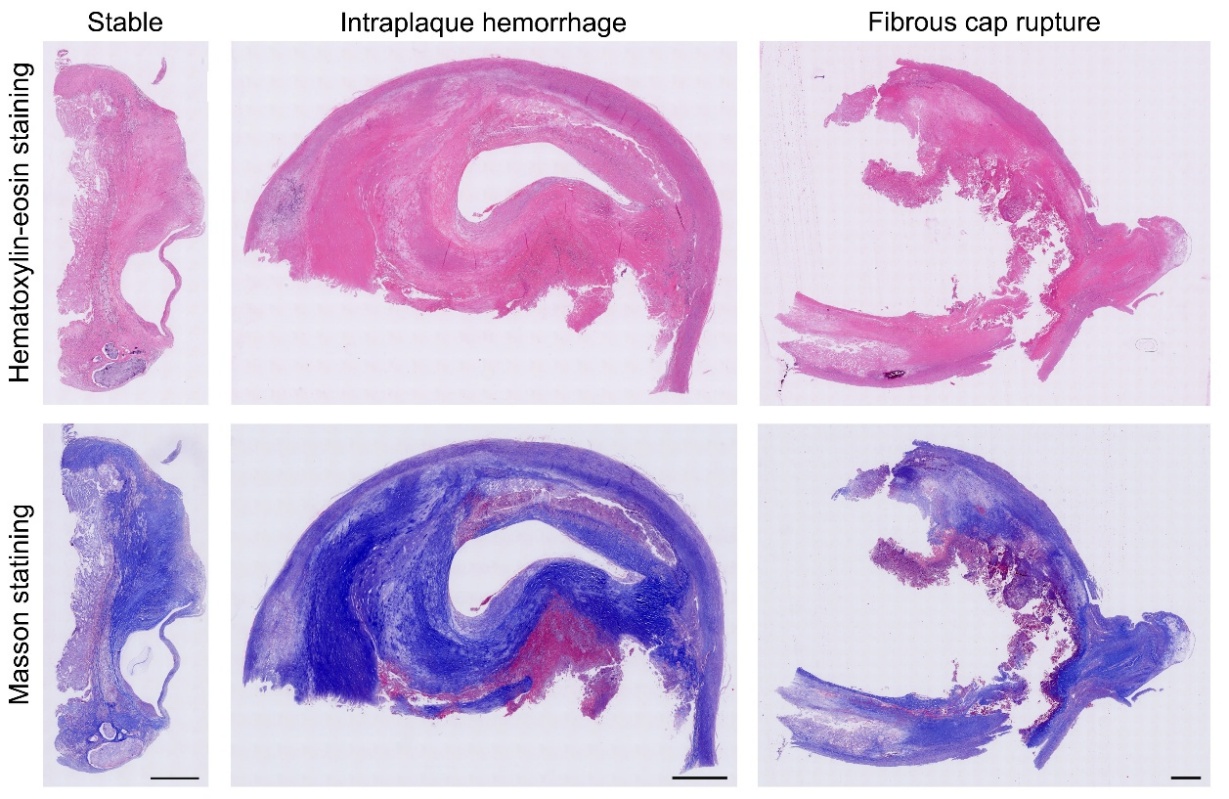


**Figure S9** Representative histological images of stable, IPH, and FCR, stained by H&E and Masson. Scale bar = 1 mm.

**Reference**

1. Hellings WE, Peeters W, Moll FL, Piers SR, van Setten J, Van der Spek PJ, de Vries JP, Seldenrijk KA, De Bruin PC, Vink A, Velema E, de Kleijn DP, Pasterkamp G. Composition of carotid atherosclerotic plaque is associated with cardiovascular outcome: A prognostic study. *Circulation*. 2010;121:1941-1950

2. Verhoeven BA, Velema E, Schoneveld AH, de Vries JP, de Bruin P, Seldenrijk CA, de Kleijn DP, Busser E, van der Graaf Y, Moll F, Pasterkamp G. Athero-express: Differential atherosclerotic plaque expression of mrna and protein in relation to cardiovascular events and patient characteristics. Rationale and design. *European journal of epidemiology*. 2004;19:1127-1133

3. Redgrave JN, Lovett JK, Gallagher PJ, Rothwell PM. Histological assessment of 526 symptomatic carotid plaques in relation to the nature and timing of ischemic symptoms: The oxford plaque study. *Circulation*. 2006;113:2320-2328

4. Crescitelli R, Lasser C, Lotvall J. Isolation and characterization of extracellular vesicle subpopulations from tissues. *Nature protocols*. 2021;16:1548-1580

5. Jingushi K, Uemura M, Ohnishi N, Nakata W, Fujita K, Naito T, Fujii R, Saichi N, Nonomura N, Tsujikawa K, Ueda K. Extracellular vesicles isolated from human renal cell carcinoma tissues disrupt vascular endothelial cell morphology via azurocidin. *International journal of cancer*. 2018;142:607-617

6. Xu X, Wang D, Han Z, Wang B, Gao W, Fan Y, Li F, Zhou Z, Gao C, Xiong J, Zhou S, Zhang S, Yang G, Jiang R, Zhang J. A novel rat model of chronic subdural hematoma: Induction of inflammation and angiogenesis in the subdural space mimicking human-like features of progressively expanding hematoma. *Brain research bulletin*. 2021;172:108-119

7. Li L, Li F, Bai X, Jia H, Wang C, Li P, Zhang Q, Guan S, Peng R, Zhang S, Dong JF, Zhang J, Xu X. Circulating extracellular vesicles from patients with traumatic brain injury induce cerebrovascular endothelial dysfunction. *Pharmacological research*. 2023;192:106791

8. Kozomara A, Griffiths-Jones S. Mirbase: Annotating high confidence micrornas using deep sequencing data. *Nucleic acids research*. 2014;42:D68-73

9. Friedlander MR, Mackowiak SD, Li N, Chen W, Rajewsky N. Mirdeep2 accurately identifies known and hundreds of novel microrna genes in seven animal clades. *Nucleic acids research*. 2012;40:37-52

10. Robinson MD, McCarthy DJ, Smyth GK. Edger: A bioconductor package for differential expression analysis of digital gene expression data. *Bioinformatics*. 2010;26:139-140

11. Zhou J, Wu Z, Hu J, Yang D, Chen X, Wang Q, Liu J, Dou M, Peng W, Wu Y, Wang W, Xie C, Wang M, Song Y, Zeng H, Bai C. High-throughput single-ev liquid biopsy: Rapid, simultaneous, and multiplexed detection of nucleic acids, proteins, and their combinations. *Science advances*. 2020;6

12. Osteikoetxea X, Sodar B, Nemeth A, Szabo-Taylor K, Paloczi K, Vukman KV, Tamasi V, Balogh A, Kittel A, Pallinger E, Buzas EI. Differential detergent sensitivity of extracellular vesicle subpopulations. *Organic & biomolecular chemistry*. 2015;13:9775-9782

13. Li P, Hong J, Liang C, Li Y, Gao L, Wu L, Yao R, Zhang Y. Endothelial cell-released extracellular vesicles trigger pyroptosis and vascular inflammation to induce atherosclerosis through the delivery of hif1a-as2. *FASEB journal : official publication of the Federation of American Societies for Experimental Biology*. 2023;37:e22942

14. Jiang F, Chen Q, Wang W, Ling Y, Yan Y, Xia P. Hepatocyte-derived extracellular vesicles promote endothelial inflammation and atherogenesis via microrna-1. *Journal of hepatology*. 2020;72:156-166
